# A Regional Mapping of Articular Cartilage Integrity and Biphasic Properties in Healthy and Osteoarthritic Trapeziometacarpal Joints

**DOI:** 10.1007/s10439-025-03726-x

**Published:** 2025-04-02

**Authors:** Lizzie Walker, Hui Li, Nathan Buchweitz, Daniel Gordon, Shangping Wang, Dane Daley, Hai Yao, Yongren Wu

**Affiliations:** 1 Department of Bioengineering, Clemson University, Clemson, SC USA; 2 Department of Orthopaedics and Physical Medicine, Medical University of South Carolina, 68 President Street, BEB 203, Charleston, SC 29425 USA

**Keywords:** Trapeziometacarpal osteoarthritis, Carpometacarpal osteoarthritis, Articular cartilage, Biphasic properties, Anterior oblique ligament, Volar ligament complex

## Abstract

**Purpose:**

To identify the mechanical and morphological regional changes of articular cartilage on the first metacarpal and trapezium and their association with attenuation of the volar ligament complex (VLC) during trapeziometacarpal (TMC) osteoarthritis (OA) progression.

**Methods:**

Twenty-four fresh-frozen female cadaveric TMCs were separated into (1) younger healthy/early-stage osteoarthritic, (2) elder healthy/early-stage osteoarthritic, and (3) advanced-stage osteoarthritic groups based on age and Eaton-Littler grading. Metacarpal and trapezium surfaces were split into six regions. Microindentation testing was performed to characterize the biphasic properties of each region. Light imaging, scanning electron microscopy/energy dispersive spectroscopy, and magnetic resonance imaging were performed to assess cartilage integrity and identify wear patterns.

**Results:**

The volar ulnar region of the metacarpal, along with the volar central and volar ulnar regions of the trapezium, had a higher equilibrium modulus in the advanced-stage OA specimens. SEM/EDS revealed these regions to be fully eburnated in most cases. MRI revealed eburnation, as well as the degeneration and/or detachment of the beak ligament portion of the VLC. In the advanced-stage OA TMCs, an increased equilibrium modulus in the volar portion of the trapezium correlates to attenuated VLC stiffness from our previous study.

**Conclusion:**

A regional pattern in articular cartilage degeneration, including changes in biphasic properties evidenced by an increased equilibrium modulus, during TMC OA progression is evident with the most significant changes occurring in the volar ulnar region of the metacarpal and volar central and volar ulnar regions of the trapezium. These changes in articular cartilage can be correlated to an attenuated VLC.

**Supplementary Information:**

The online version contains supplementary material available at 10.1007/s10439-025-03726-x.

## Introduction

The trapeziometacarpal (TMC) joint is a biconcave saddle shaped joint between the trapezium and first metacarpal. This joint allows for a wide range of motions including flexion/extension and abduction/adduction and is also responsible for human’s ability to perform important functional tasks such as key pinch and jar twist motions. This wide range of motion comes at a cost, however, as the TMC joint is the most common site for osteoarthritis (OA) in the upper limb, especially in postmenopausal females who are disproportionally affected by this disease compared to their male counterparts of the same age [1–7]. TMC OA is often characterized by narrowing of joint space, thinning and eburnation of articular cartilage, osteophyte formation, and subluxation of the metacarpal.

**Fig. 1:**
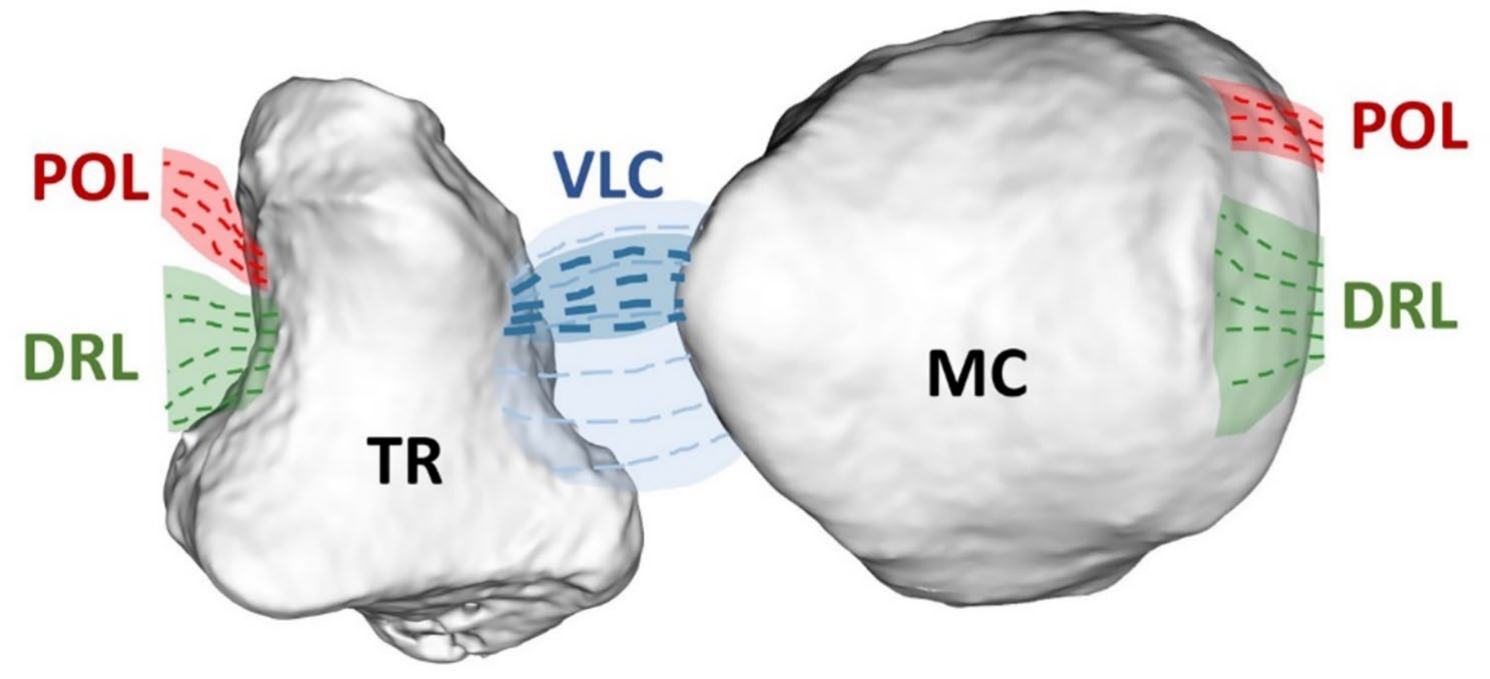
Illustration of TMC joint that has been opened to reveal the metacarpal and trapezium surfaces, as well as ligament insertions and origins

The TMC joint has limited bony stability and relies heavily on ligaments and muscles to act as its stabilizers [8]. The dorsoradial ligament (DRL), posterior oblique ligament (POL), and volar ligament complex (VLC) [including the superficial anterior oblique ligament (AOL) and beak ligament (BL)] have all been identified as key stabilizers for the joint [9–14] (Fig. 1). Our previous research has shown the VLC to experience attenuated mechanical properties and lose structural integrity at the enthesis of the metacarpal in osteoarthritic joints [15]. Other studies have demonstrated that a detached or degenerated beak ligament can lead to an increase in dorsal translation of the metacarpal, causing articular cartilage wear within the joint [14].

Both qualitative and quantitative studies have been performed to understand the properties of articular cartilage within the TMC joint. Research has found that metacarpal cartilage degeneration begins in the palmar/volar ulnar region, adjacent to the beak ligament, and spreads dorsoradially while trapezium cartilage degeneration begins in the palmar/volar central region and spreads centrifugally as OA progresses [12]. This is further supported by a study that found a decrease in hydroxyproline and proteoglycan content and an increase in water content of cartilage in osteoarthritic TMC joints with the volar sides of the metacarpal and trapezium being more severe [16]. Additionally, scanning electron microscopy (SEM) has revealed that intermediate disease, characterized by chondromalacia, was largely found in volar portions of the joint [17]. These same volar regions were found to be fully eburnated in cases of advanced-stage OA [17]. A study performed on healthy TMC joints divided the metacarpal and trapezium each into nine different regions and found there to be no difference in cartilage stiffness between regions, but that the metacarpal was overall stiffer than the trapezium [18]. Additionally, it has been reported that an overall decrease in aggregate modulus and permeability can be seen in osteoarthritic TMC cartilage, however this study did not specify which regions these properties were coming from [19].

The objective of this study is to investigate the changes in cartilage wear patterns and material properties of individual regions within the metacarpal and trapezium articular cartilage surfaces that occur throughout OA progression and correlate biphasic properties of TMC articular cartilage to TMC ligament mechanical properties within the same joint, which has not been previously studied to the best of our knowledge. A histopathology study shows thinning of the articular cartilage on the metacarpal, adjacent to the beak ligament insertion, in conjunction with beak ligament degeneration and detachment [20]. During functional tasks such as key pinch, contact areas are predominantly located in the volar, volar/ulnar, and or central region of the metacarpal and trapezium and spread dorsally when the task becomes dynamic [14, 21]. When the beak ligament is cut in a healthy joint, contact areas that were once localized to the volar compartments, spread dorsally, producing a pattern similar to what is seen in osteoarthritic joints [14]. Because of this, we believe that we will see a deterioration of articular cartilage, primarily in the volar ulnar region of the metacarpal and volar central region of the trapezium, made evident by our microindentation mechanical testing and advanced imaging modalities. We believe articular cartilage deterioration seen in advanced-stage OA samples can be correlated to VLC attenuation in the same samples from our previous study [15].

## Materials and Methods

### Specimen Preparation

Twenty four fresh-frozen female cadaver hands ranging in age from 28 to 97 were obtained through an organ procurement organization (approved by Institutional Review Board for Human Research at the Medical University of South Carolina). Females were exclusively chosen for this study as aging, postmenopausal females are the most susceptible group for TMC OA [1–7, 15]. Specimens were segmented either proximal or distal to the elbow and stored in a − 20 °C freezer. Lateral and Robert’s view radiographs were obtained for each specimen and blindly graded by our clinical collaborator (A.C. and D.D.) based on the Eaton-Littler classification system [22]. The hand specimens were organized into three experiment groups: (1) younger specimens with early-stage or no OA (n = 8, < 45 year, Eaton-Littler Grade 0–II), (2) elder specimens with early-stage or no OA (n = 8, > 45 year, Eaton-Littler Grade 0–II), and (3) any specimens with advanced-stage osteoarthritic changes (n = 8, no age requirement, Grade III–IV). Six specimens per group were randomly selected for mechanical testing, and the remaining two specimens per group were used for scanning electron microscopy.

**Fig. 2:**
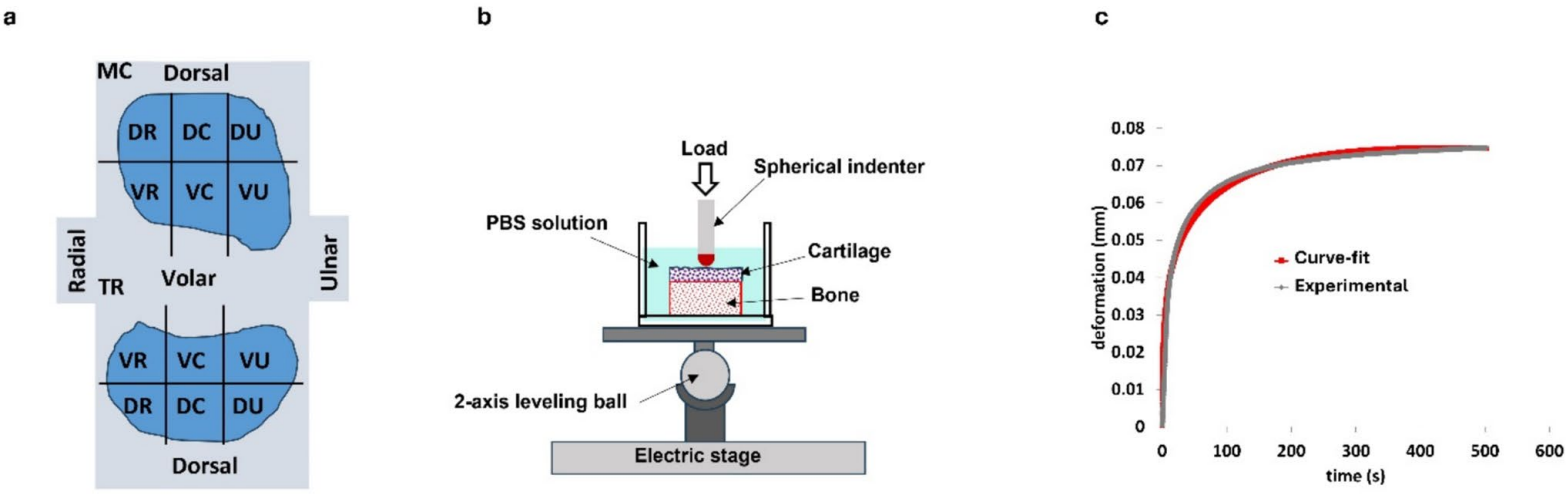
Experiment setup: **a** schematic of metacarpal (MC) and trapezium (TR) regions, **b** schematic of microindentation setup, **c** typical deformation vs. time plot from creep experiment

The TMC joint was excised from the thumb following guidelines from previously published studies [11, 15, 23]. Once free, the TMC joint was split coronally, resulting in a volar and dorsal half from both the first metacarpal and the trapezium. These halves were then split into three regions: radial, central, and ulnar. This resulted in 6 regions each for the metacarpal and trapezium articulating surfaces: dorsal radial (DR), dorsal central (DC), dorsal ulnar (DU), volar radial (VR), volar central (VC), and volar ulnar (VU) (Fig. 2a). Samples were wrapped in plastic wrap and PBS-soaked gauze and stored in a − 20 °C freezer until use to avoid desiccation. All samples were limited to five or less freeze/thaw cycles prior to testing [24–26].

### Microindentation Mechanical Testing

Microindentation was performed using an Anton Paar Indentation Tester (UNHT^3^, Anton Paar) with a ruby ball indenter probe (R = 0.5 mm). The specimens were glued to the bottom of a 35 mm glass bottom dish and submerged in PBS to avoid desiccation during the testing process (Fig. 2b). A preload of 100 µN was applied for 400 s before a 5000 µN creep load was applied for 600 s. These times were chosen based on previous benchmark testing that showed equilibrium could be reached by these time points. The responding deformation was recorded. Responding deformation was between 50 and 80 µm for healthy cartilage and 20 and 40 µm for eburnated bone. Articular cartilage thickness for each sample was recorded using a light microscope with an attached, calibrated camera in conjunction with a photo software (AmScope) to view and measure the cross section of cartilage. The applied force and responding deformation from the creep experiments, probe radius, and cartilage thickness were used as inputs to calculate equilibrium contact modulus and hydraulic permeability based on the curve-fitting of deformation vs. time (Fig. 2c) [27]. This model assumes a Poisson’s ratio of zero [27].

### Porosity and Water Content Calculations

Following microindentation, cartilage was separated from the underlying subchondral bone and immediately weighed for wet and PBS weight on a precision analytical balance scale with a scale division of 0.1 mg. Wet weight is calculated as the weight of the fresh cartilage before any drying out has occurred. PBS weight is calculated as the weight of the fresh cartilage when placed in 1X PBS. Following wet and PBS weight calculations, samples were placed in a 65 °C oven for three days. Dry weight was taken immediately upon removal from the oven as the weight of the now fully desiccated sample.

Porosity was calculated using the following equation:

1 $${\varnothing }^{w}=\frac{{W}_{wet}-{W}_{dry}}{{W}_{wet}-{W}_{PBS}}*\frac{{\rho }_{PBS}}{{\rho }_{w}}$$

Water content was calculated using the following equation:

2 $$WC= \frac{{W}_{wet}-{W}_{dry}}{{W}_{wet}} x 100$$

where ${W}_{wet}$ and ${W}_{dry}$ denote the wet weight and dry weight, respectively, ${W}_{PBS}$ denotes the weight of the specimen when measured in PBS, and $\frac{{\rho }_{PBS}}{{\rho }_{w}}$ denotes the relative density of PBS to water [28–30].

### Light Imaging

Prior to splitting the metacarpal and trapezium surfaces into separate regions, trinocular stereo zoom microscope (AmScope) was used to photograph the entire articular surface at 0.8× to view cartilage wear and VLC attachment sites on the metacarpal.

### Magnetic Resonance Imaging

Prior to any dissection, TMC joints were imaged utilizing a 7T Magnetic Resonance Imaging (MRI) machine with a Proton Dense-T2-MSME sequence where field of view was set to 38.4 mm × 28.8 mm, matrix was 192 × 144, resolution was 0.2 mm × 0.2 mm × 0.3 mm, with 80 slices, a time repetition of 3300 ms, and a time of echo of 12 and 24 ms. Segmentation and articular cartilage volume and surface area calculations were performed using Amira (version 6.0.1). These calculations were performed for each of the six regions on both the metacarpal and trapezium articular cartilage surfaces.

### Scanning Electron Microscopy and Energy Dispersive Spectroscopy

Scanning electron microscopy (SEM) (S-3700N; Hitachi) coupled with energy dispersive spectroscopy (EDS) (AZtec; Oxford Instruments) was used to observe cartilage integrity and eburnation patterns in each of the six regions of the metacarpal and trapezium articular cartilage for the three experiment groups. Specimens were placed in a desiccator for two days until fully dehydrated. Prior to imaging, they were then gold-sputtered at 50 mA for 30 s. Backscattered electron (BSE) and secondary electron (SE) images were taken at 10 kV at a magnification of 100x. The mapping function of the EDS was utilized to view the elemental composition of the articulating surface. Carbon represents the presence of articular cartilage, while phosphorus and calcium represent an eburnated articulating surface.

### Statistical Analysis

The effects of OA group (younger healthy/early-stage OA; elder healthy/early-stage OA; advanced-stage OA) and region (DR, DC, DU, VR, VC, VU on metacarpal and trapezium) on articular cartilage thickness, permeability, and equilibrium modulus outcomes were evaluated. Linear mixed-effects models were fit to each outcome, with OA group and region or ligament indicators modeled as fixed effects, and donor information included as a random effect to account for within-donor correlation. Effect of hand side (left or right) was initially modeled, but no hand side dependence was found; therefore, it was removed. Interactions can be found in Table 1 in Online Resource 1. Where significant differences were seen, pairwise comparisons were made, incorporating a Bonferroni *P*-value adjustment to account for multiplicity error. Pearson correlation coefficients were calculated based on the data reported in this study and our previously reported TMC ligament data that comes from the same cohort of female specimens [15]. All statistical inferences are derived from the fitted mixed-effects model. Statistical differences are reported as *P < *0.05*.* Data presented as mean ± standard error. Statistical analyses were performed in SPSS (version 28.0.1.0; IBM).

## Results

### Tissue Thickness

Articular cartilage was thinnest overall in the advanced-stage OA specimens compared to the younger (*P* < 0.001) and elder (*P* < 0.001) healthy/early-stage OA groups. Additionally, the volar ulnar region of the metacarpal had the thinnest cartilage overall when compared to the volar central (*P* = 0.038) and volar ulnar (*P* = 0.013) regions of the metacarpal.

**Fig. 3:**
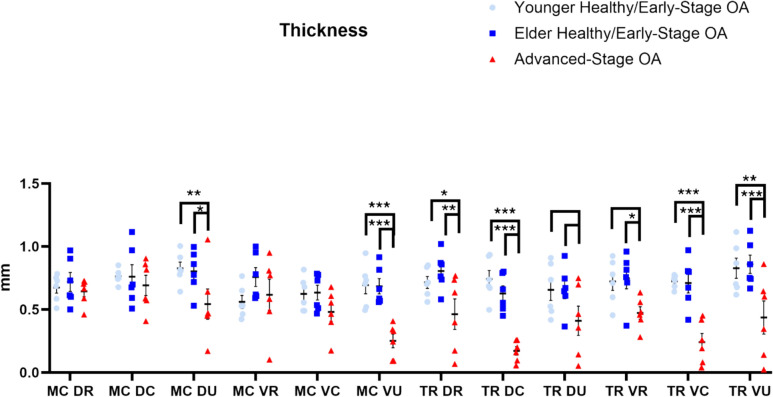
Articular cartilage thickness results. *** denotes *P* < 0.001, ** denotes *P* < 0.01, * denotes *P* < 0.05, blank bar denotes weak trend with 0.05 ≤ *P* ≤ 0.1

Articular cartilage was thinner in the dorsal ulnar (0.54 ± 0.12 mm) and volar ulnar (0.25 ± 0.05 mm) regions of the metacarpal in advanced-stage OA TMC joints when compared to those regions in the younger (DU: 0.83 ± 0.05 mm, *P* = 0.009; VU: 0.69 ± 0.07 mm, *P* < 0.001) and elder (DU: 0.80 ± 0.07 mm, *P* = 0.018; VU: 0.68 ± 0.06 mm, *P* < 0.001) healthy/early-stage OA groups. Thinner articular cartilage was also seen in the dorsal radial (0.46 ± 0.12 mm), dorsal central (0.17 ± 0.03 mm), volar central (0.24 ± 0.07 mm), and volar ulnar (0.44 ± 0.13 mm) regions of the trapezium in advanced-stage OA TMC joints when compared to those regions in the younger (DR: 0.72 ± 0.05 mm, *P* = 0.05; DC: 0.74 ± 0.07 mm, *P* < 0.001; VC: 0.72 ± 0.02 mm, *P* < 0.001; VU: 0.83 ± 0.08 mm, *P* = 0.001) and elder (DR: 0.81 ± 0.06 mm, *P* = 0.01; DC: 0.63 ± 0.06 mm, *P* < 0.001; VC: 0.71 ± 0.08 mm, *P* < 0.001, VU: 0.86 ± 0.07 mm, *P* < 0.001) healthy/early-stage OA groups. Additionally, thinner cartilage was seen in the volar radial region of the trapezium in advanced-stage OA TMC joints (0.47 ± 0.05 mm) when compared to the elder healthy/early-stage OA group (0.75 ± 0.08 mm, *P* = 0.041) (Fig. 3).

A weak trend also shows thinner cartilage in the dorsal ulnar (0.41 ± 0.12 mm) region of the trapezium in advanced-stage OA TMC joints compared to the younger (0.66 ± 0.09 mm, *P* = 0.088) and elder (0.66 ± 0.08 mm, *P* = 0.086) healthy/early-stage OA TMC joints, as well as in the volar radial (0.47 ± 0.05 mm) region of the trapezium in advanced-stage OA specimens when compared to the elder healthy/early-stage OA group (0.86 ± 0.08 mm, *P* = 0.083) (Fig. 3).

In the advanced-stage OA group, thinner articular cartilage was found in the volar ulnar region of the metacarpal compared to the dorsal radial (*P* = 0.017), dorsal central (*P* = 0.001), and volar radial (*P* = 0.005) regions of the metacarpal. Additionally, the dorsal central region of the trapezium had thinner cartilage than the dorsal radial (*P* = 0.002), dorsal central (*P* < 0.001), dorsal ulnar (*P* = 0.02), and volar radial (*P* < 0.001) regions of the metacarpal. Finally, the volar central region of the trapezium had thinner cartilage than dorsal radial (*P* = 0.012), dorsal central (*P* < 0.001), and volar radial (*P* = 0.003) regions of the metacarpal. All sample means and standard errors were listed in Table 2 in Online Resource 1. Detailed model-based comparisons of articular cartilage thickness were listed in Tables 3 and 4 in Online Resource 1.

### Mechanical Properties

Equilibrium modulus values were higher overall in the advanced-stage OA group (0.66 ± 0.07 MPa) when compared to the younger (0.45 ± 0.07 MPa, *P* = 0.09) and elder (0.41 ± 0.07 MPa, *P* = 0.028) healthy/early-stage groups. There were no significant differences when comparing only regions.

**Fig. 4:**
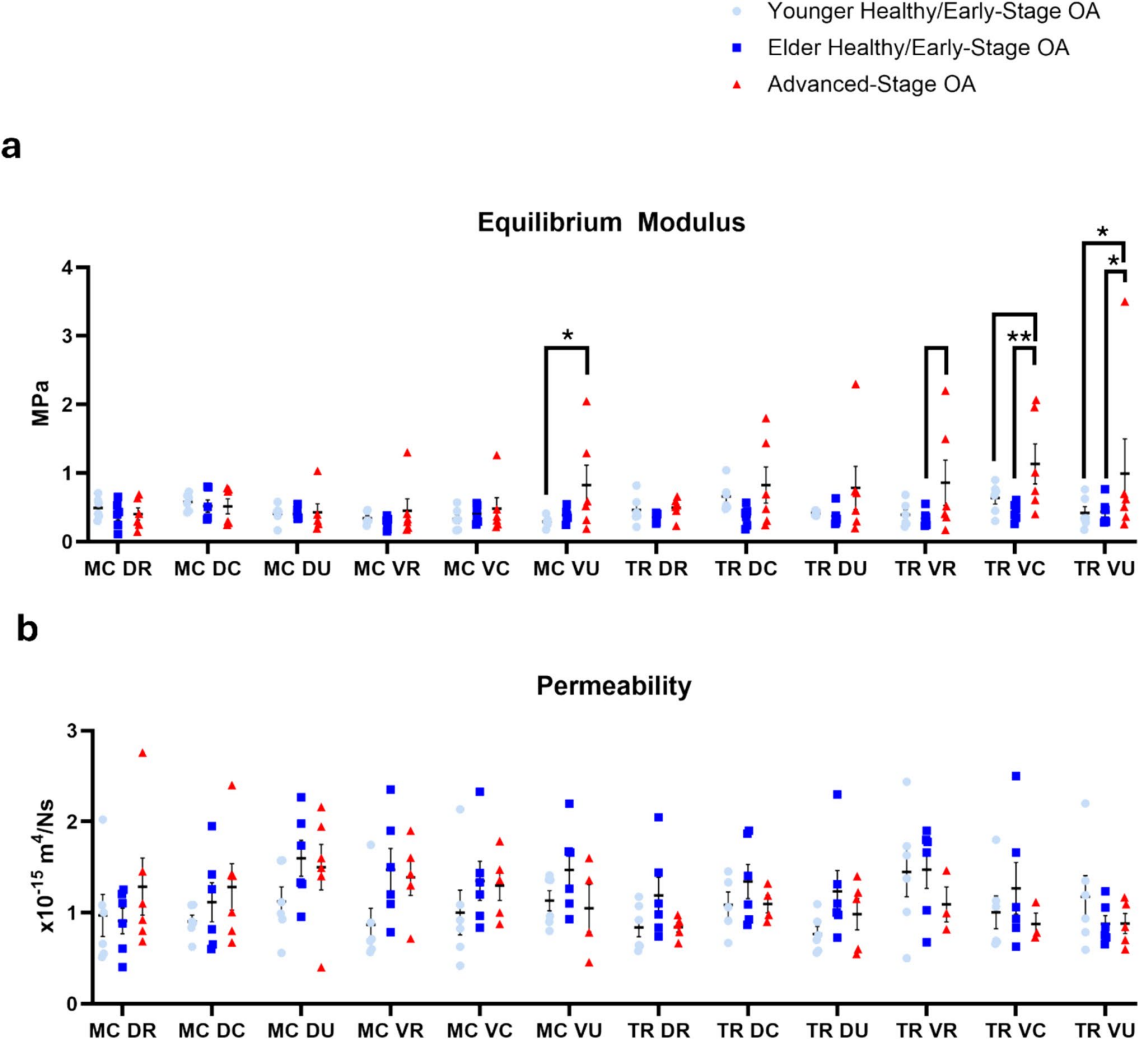
Biphasic properties: **a** equilibrium modulus, **b** permeability. ** denotes *P* < 0.01, * denotes *P* < 0.05, blank bar denotes weak trend with 0.05 ≤ *P* ≤ 0.1

Specifically, equilibrium modulus was higher in the volar ulnar (0.83 ± 0.29 MPa, *P = *0.024) region of the metacarpal, as well as the volar central (1.13 ± 0.29 MPa, *P = *0.092) and volar ulnar (0.99 ± 0.51 MPa, *P* = 0.037) regions of the trapezium in the advanced-stage OA specimens compared to the younger healthy/early-stage OA group (MC VU: 0.29 ± 0.32 MPa, TR VC: 0.64 ± 0.09 MPa, TR VU: 0.42 ± 0.09 MPa). Similarly, the volar radial (0.86 ± 0.33 MPa, *P* = 0.093), volar central (1.13 ± 0.29 MPa, *P* = 0.008), and volar ulnar (0.99 ± 0.51 MPa, *P* = 0.057) regions of the trapezium had a higher equilibrium modulus in the advanced-stage OA specimens compared to those in the elder healthy/early-stage OA group (VR: 0.34 ± 0.45 MPa; VC: 0.44 ± 0.05 MPa; VU: 0.43 ± 0.08 MPa) (Fig. 4). No significant changes in permeability were observed. All sample means and standard errors were listed in Table 4 in Online Resource 1. Detailed model-based comparisons of the biphasic properties for each region were listed in Tables 5 and 6 in Online Resource 1.

### Ligament Stiffness vs. Articular Cartilage Biphasic Properties Correlation

Within the advanced-stage OA TMC joints, a significant positive correlation was seen between permeability values in the volar half of the trapezium from this study and VLC stiffness from our previous study [15] (r^2^ = 0.61, *P* = 0.02). Similarly, a significant negative correlation was seen between equilibrium modulus of the volar half of the trapezium and VLC stiffness (r^2^ = 0.50, *P* = 0.005). No significance was seen between biphasic properties of the metacarpal and VLC stiffness. All correlation data can be found in Tables 7 and 8 in Online Resource 1.

### Porosity and Water Content

Porosity values were lowest overall in the younger healthy/early-stage OA group (0.76 ± 0.02) compared to the elder healthy/early-stage OA (0.80 ± 0.01, *P* = 0.359) and advanced-stage OA (0.82 ± 0.02, *P* = 0.073) groups. There were no significant differences when comparing only regions.

**Fig. 5:**
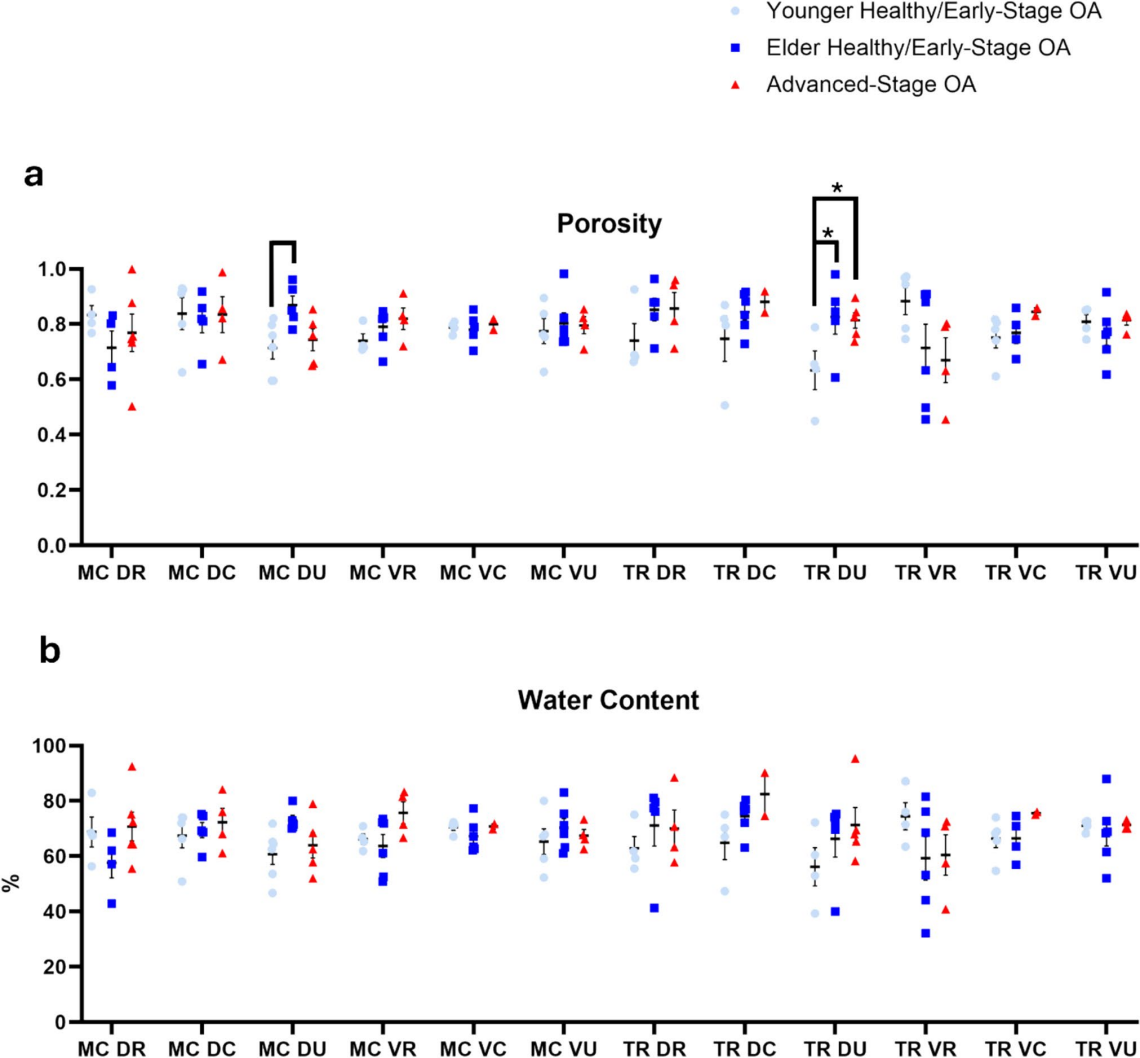
**a** porosity, **b** water content. * denotes *P* < 0.05, blank bar denotes weak trend with 0.05 ≤ *P* ≤ 0.1

When observing changes in porosity within regions of different disease groups, an increase in the dorsal ulnar region of the trapezium in the elder healthy/early-stage OA group (0.83 ± 0.06, *P* = 0.032) and advanced-stage OA group (0.81 ± 0.03, *P* = 0.048) was observed compared to the younger healthy/early-stage OA group (0.63 ± 0.07). Additionally, a weak trend showed an increase in porosity in the dorsal ulnar (0.87 ± 0.03, *P* = 0.07) region of the metacarpal in the elder healthy/early-stage group compared to the younger group (0.71 ± 0.04). No significant changes in water content were observed (Fig. 5). All sample means and standard errors were listed in Table 2 in Online Resource 1. Detailed model-based comparisons of porosity and water content for each region were listed in Tables 9 and 10 in Online Resource 1.

### Tissue Imaging

#### Light Imaging

In the younger healthy/early-stage TMC joints, partial articular cartilage wear on the metacarpal surface was present in roughly 17% of specimens in the volar central region and 50% of specimens in the volar ulnar region, mostly concentrated adjacent to the intraarticular attachment of the beak ligament. Partial articular cartilage wear on the trapezium was observed in roughly 17% of specimens in the dorsal central and volar central regions.

The elder healthy/early-stage specimens demonstrated partial articular cartilage wear on the metacarpal in roughly 17% of specimens in the volar radial region, 50% of specimens in the volar central region, and 100% of specimens in the volar ulnar region. Within this disease group, partial articular cartilage wear on the trapezium was seen in roughly 17% of specimens in the dorsal radial region, 50% in the dorsal central region, 17% in the volar radial region, 50% in the volar central region, and roughly 67% in the volar ulnar region.

**Fig. 6:**
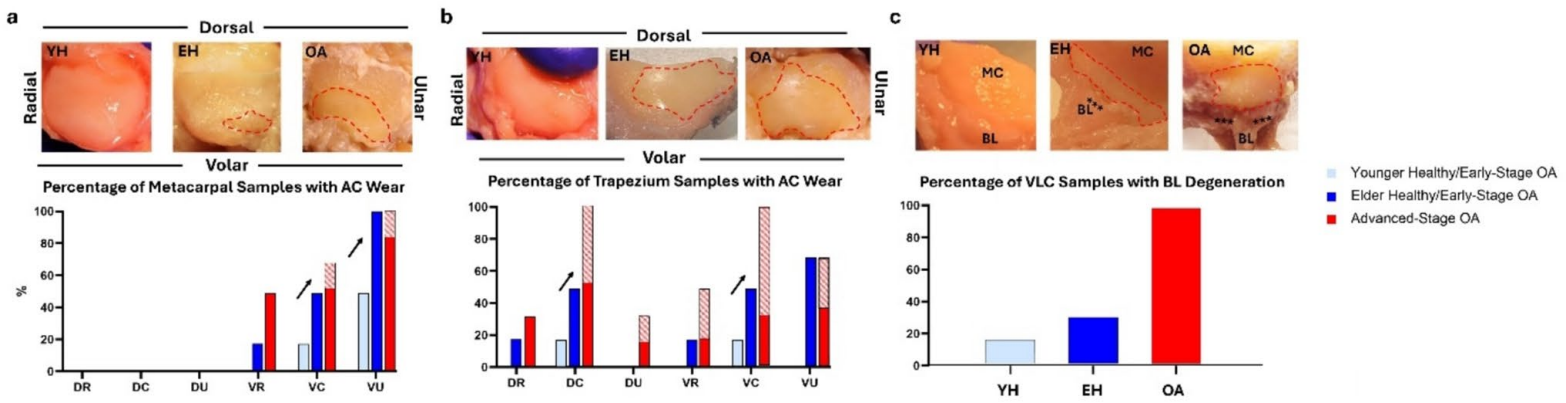
Cartilage wear and ligament detachment patterns: **a** percentage of TMC joints with metacarpal wear along with corresponding light imaging, **b** percentage of TMC joints with trapezium wear along with corresponding light imaging, **c** percentage of TMC joints with BL degeneration within the VLC along with corresponding light imaging. Solid bar graph denotes partial cartilage degeneration, striped bar denotes full cartilage eburnation, ***denotes BL degeneration and/or detachment

Within the advanced-stage OA group, 50% of specimens demonstrated partial articular cartilage wear on the volar central surface of the metacarpal while 17% of specimens demonstrated full cartilage eburnation in this region. Similarly, in the volar ulnar region of the metacarpal, 83% of specimens demonstrated partial articular cartilage wear while 17% demonstrated full cartilage eburnation. Within the trapezium surface of the advanced-stage TMC joints, 33% of specimens demonstrated partial articular cartilage wear in the dorsal radial region, 50% with partial wear and 50% with full eburnation in the dorsal central, 17% with partial wear and 17% with full eburnation in the dorsal ulnar, 17% with partial wear and 33% with full eburnation in the volar radial, 33% with partial eburnation and 67% with full eburnation in the volar central, and 33% with partial wear and 33% with full eburnation in the volar ulnar region (Fig. 6a and b).

Additionally, when observing VLC integrity, 17% of younger healthy/early-stage, 33% of the elder healthy/early-stage OA, and 100% of the advanced-OA stage specimens showed beak ligament detachment. In the elder healthy/early-stage OA group and advanced-stage OA group, this detachment was seen in correlation to sever cartilage wear and/or eburnation of the area adjacent to the insertion (Fig. 6c).

### MRI

**Fig. 7:**
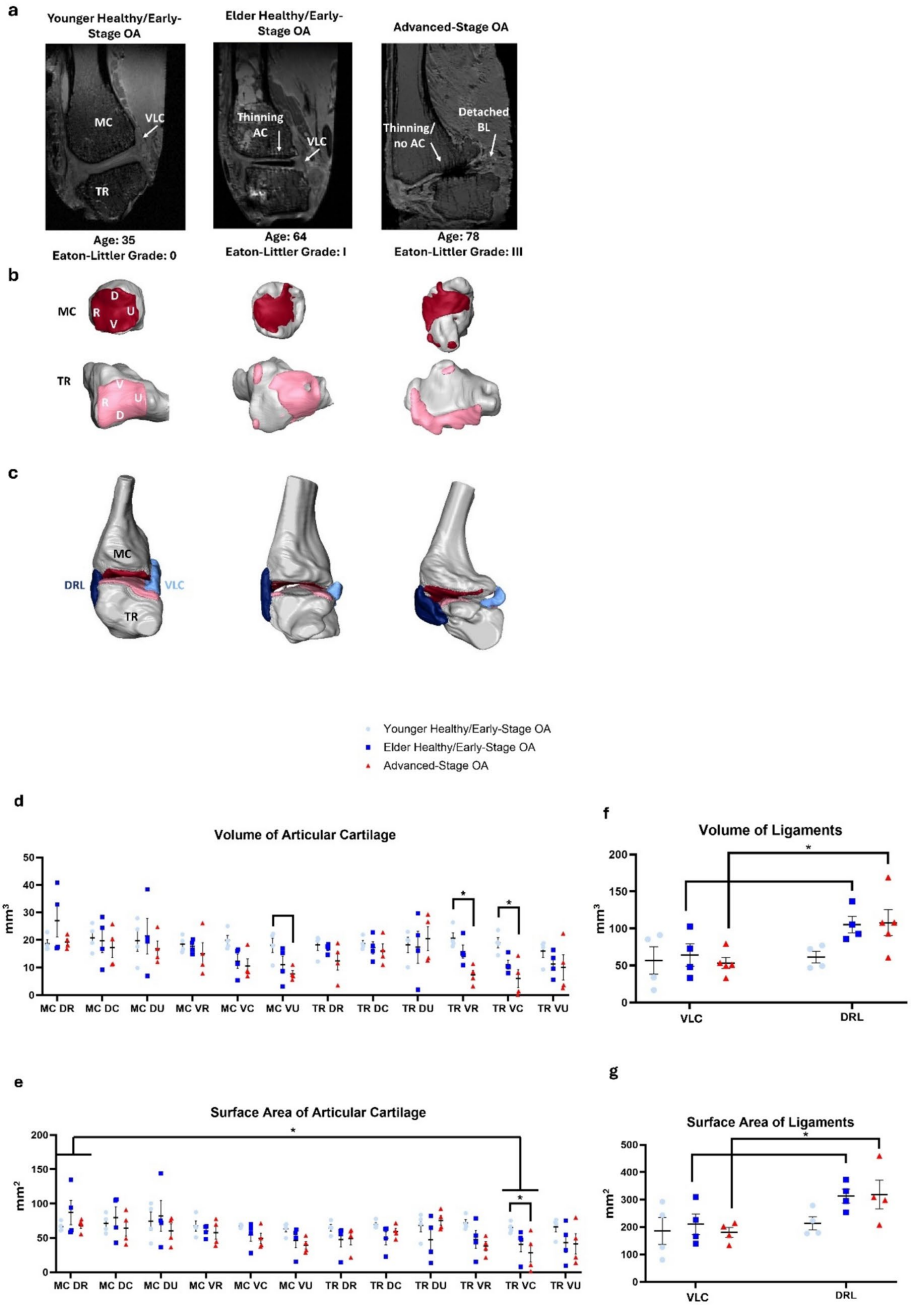
MRI of TMC joint: **a** single frame of MRI of representative sample from each disease group, **b** 3D segmentation showing articular cartilage wear pattern of metacarpal (red) and trapezium (pink), **c** 3D segmentation of entire TMC joint including articular cartilage, DRL (dark blue) and VLC (light blue), **d** MRI volume of articular cartilage regions, **e** MRI surface area of articular cartilage regions, **f** MRI volume of VLC and DRL, **g** MRI surface area of VLC and DRL. ** denotes *P* < 0.01, * denotes *P* < 0.05, blank bar denotes weak trend with 0.05 ≤ *P* ≤ 0.1

Overall, articular cartilage seemed to thin first in the volar ulnar region, adjacent to the intraarticular insertion of the beak ligament, and spread dorsoradially as OA progressed. In the trapezium, cartilage thinned first in the volar and dorsal central regions and seemed to spread centrifugally during OA progression. The VLC metacarpal insertion appeared to detach in severely diseased specimens while the DRL remained unchanged (Fig. 7a–c).

### Volume and Surface Area of Articular Cartilage

Articular cartilage volume was highest overall in the dorsal radial region of the metacarpal (21.65 ± 11.12 mm^3^), specifically when compared to the volar ulnar (12.21 ± 8.01 mm^3^, *P* = 0.004) region of the metacarpal and volar central (12.00 ± 7.86 mm^3^, *P* = 0.003) and volar ulnar (12.43 ± 6.73 mm^3^, *P* = 0.005) regions of the trapezium. There were no significant differences when comparing only disease group.

When observing changes in articular cartilage volume within regions of different disease groups, a decrease in volume of articular cartilage in the volar central (5.99 ± 3.28 mm^3^, *P* = 0.014) and volar radial (7.29 ± 1.71 mm^3^, *P* = 0.011) regions of the trapezium was seen in the advanced-stage OA specimens compared to those in the younger healthy/early-stage OA group (VC: 18.97 ± 1.86 mm^3^; VU: 15.95 ± 2.56 mm^3^). A weak trend showed a decrease in volume of articular cartilage in the volar ulnar region of the metacarpal in advanced-stage OA specimens (*P* = 0.066) compared to those in the younger healthy/early-stage OA group.

Within the elder healthy/early-stage OA group, the dorsal radial region of the metacarpal (27.04 ± 5.89 mm^3^) had a higher volume of articular cartilage than the volar central and volar ulnar regions of both the metacarpal (VC: 12.32 ± 2.58 mm^3^, *P* = 0.017; VU: 10.84 ± 3.09 mm^3^, *P* = 0.004) and trapezium (VC: 11.05 ± 1.61 mm^3^, *P* = 0.005; VU: 11.25 ± 2.36 mm^3^, *P* = 0.006) (Fig. 7d).

Articular cartilage surface area was smallest overall in the volar central region of the trapezium (44.81 ± 7.14 mm^2^) compared to the dorsal radial region of the metacarpal (74.09 ± 6.31 mm^2^, *P* = 0.30). Additionally, the surface area of the volar central region of the trapezium in the advanced-stage OA specimens (28.55 ± 13.75 mm^2^) was smaller than in the younger early/healthy-stage OA specimens (65.08 ± 3.92 mm^2^, *P* = 0.041) (Fig. 7e). All sample means and standard errors were listed in Table 2 in Online Resource 1. Detailed model-based comparisons of MRI derived measurements for articular cartilage for each region were listed in Tables 11 and 12 in Online Resource 1.

### Volume and Surface Area of Ligaments

The VLC was smaller in volume (58.07 ± 7.93 mm^3^), overall, than the DRL (91.48 ± 10.27 mm^3^, *P* = 0.014). Within the advanced-stage OA specimens, the VLC had a smaller volume (53.19 ± 9.66 mm^3^) than the DRL (107.82 ± 22.67 mm^3^, *P* = 0.020). There was also a weak trend suggesting the VLC was smaller in volume than the DRL within the elder healthy/early-stage group (*P* = 0.071) (Fig. 7f).

The VLC was also smaller in surface area (192.03 ± 19.98 mm^2^), overall, compared to the DRL (281.78 ± 23.82 mm^2^, *P* = 0.008). Within the advanced-stage OA group, the VLC was smaller in surface area (180.11 ± 18.25 mm^2^) than the DRL (318.26 ± 52.19 mm^2^, *P* = 0.016) There was also a weak trend suggesting the VLC was smaller in volume than the DRL within the elder healthy/early-stage group (*P* = 0.065) (Fig. 7g). All sample means and standard errors were listed in Table 2 in Online Resource 1. Detailed model-based comparisons of MRI derived measurements for the VLC and DRL were listed in Tables 13–16 in Online Resource 1.

### SEM/EDS

**Fig. 8:**
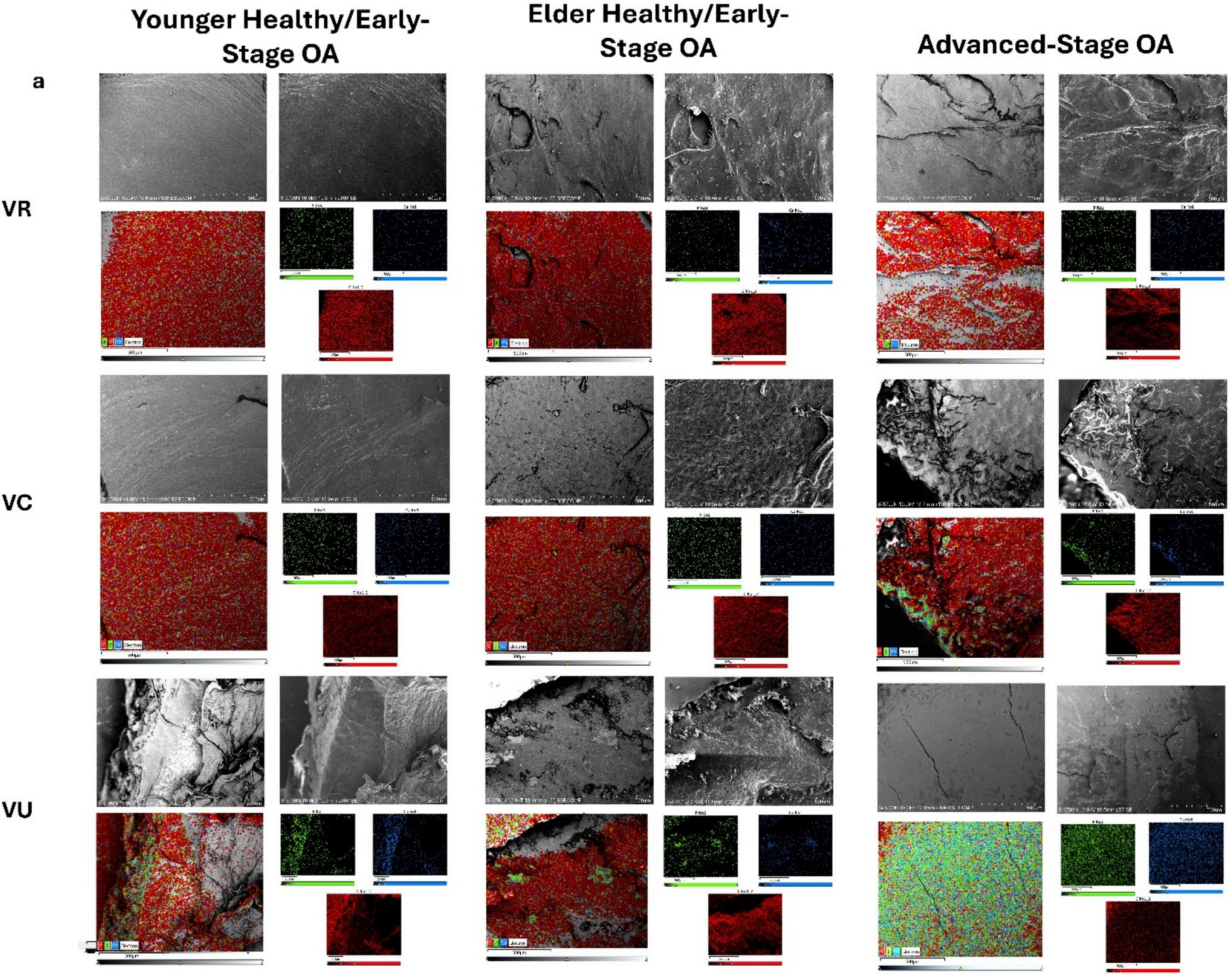

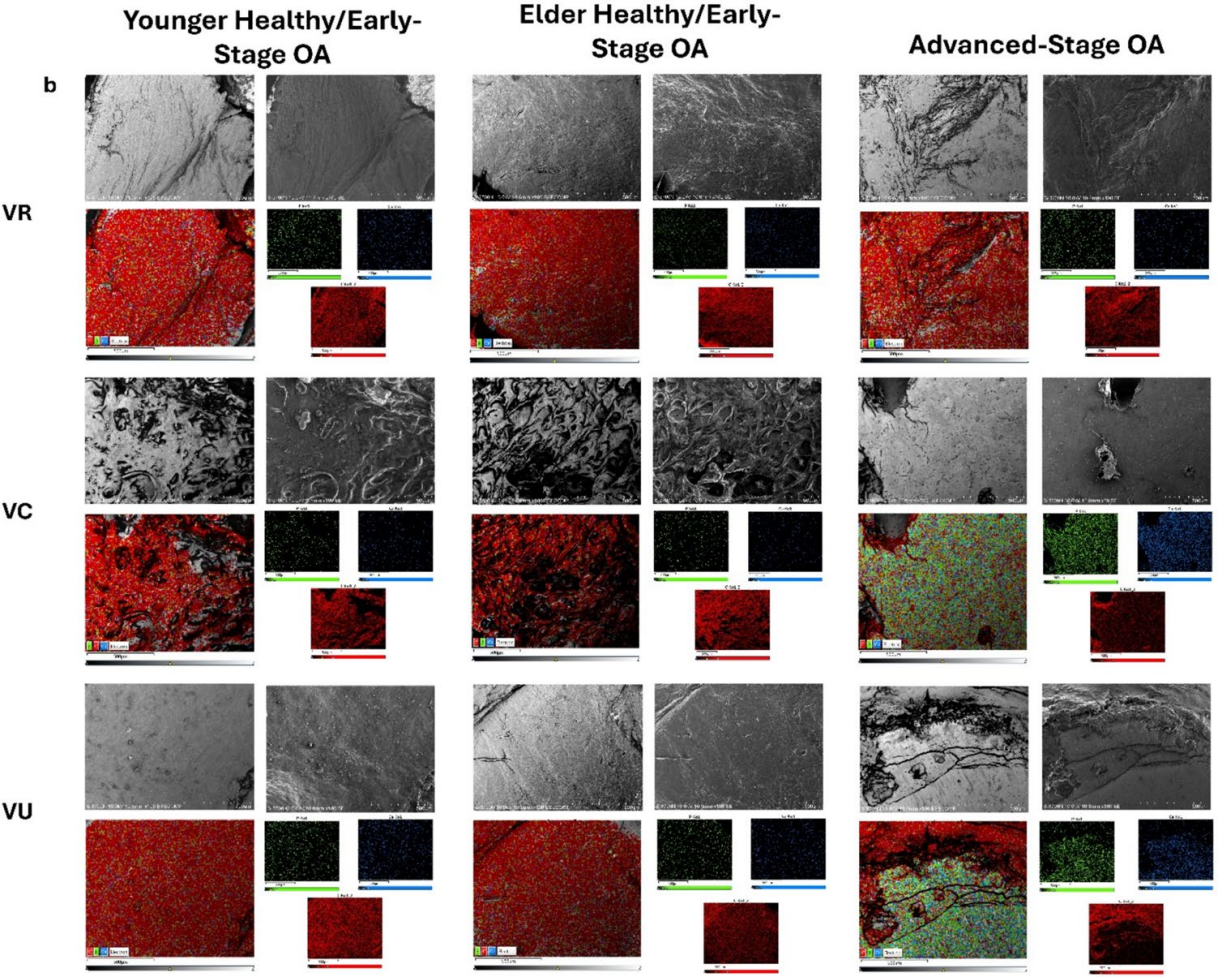
SEM/EDS images of representative samples from each disease group: **a** volar radial, volar central, and volar ulnar regions of the metacarpal, **b** volar radial, volar central, and volar ulnar regions of the trapezium. Each region contains BSE image (top left), SE image (top right), EDS layered image (bottom left), and individual EDS layers (bottom right). Red represents carbon, green represents phosphorous, and blue represents calcium. Any gray areas on the EDS layered image are due to unevenness of the sample or shadows

The younger healthy/early-stage OA specimens revealed no signs of articular cartilage eburnation in any regions. Most regions appeared as healthy cartilage, meaning the SEM images showed a smooth, uninterrupted surface topography and EDS showed an ample presence of carbon on the surface (Fig. 8) (Fig. 1, Online Resource 2). An exception to this, however, were the volar ulnar region of the metacarpal and volar central region of the trapezium where the surfaces were uneven or rough (Fig. 8).

The elder healthy/early-stage specimens also showed healthy articular cartilage in most regions, with the volar ulnar region of the metacarpal and the volar central region of the trapezium being the exceptions once again (Fig. 8). In this case, however, the cartilage was in worse shape than the previous group. The volar ulnar region of the metacarpal appeared to be characterized by a rougher surface texture and had spots of eburnation beginning to come through, as seen by the green and blue dots on the EDS images. The volar central region of the trapezium appeared even more rough and had an increase in frequency of holes within the cartilage but no sign of eburnation yet (Fig. 8).

The advanced-stage OA specimens had more areas that showed cartilage wear and/or eburnation than the two healthy groups. The volar central region of the metacarpal showed a mixture of smooth cartilage, rough cartilage, and eburnation. Both the rough cartilage and eburnated surface appeared to be at a lower depth than the smooth cartilage. The volar ulnar region of the metacarpal was fully eburnated. The SEM images were completely smooth but lacked any signs of collagen fibrils and the flowy, almost fluid nature of healthy cartilage. The EDS images proved the presence of eburnated bone based on the substantial amounts of phosphorous and calcium that were present (Fig. 8). The same trend was seen in the volar central, volar ulnar, dorsal central, and dorsal ulnar regions of the trapezium. In the volar radial and dorsal central trapezium regions, some cartilage can still be seen as the very perimeter of joint surfaces often still maintain a little bit of cartilage. Additionally, the dorsal radial region of the trapezium had the same rough surface, perforated with holes, which had been seen in the volar central regions of the younger and elder healthy/early-stage specimens (Fig. 8) (Fig. 1, Online Resource 2).

## Discussion

A significant negative correlation was seen between VLC stiffness reported in our previous paper [15] and the equilibrium modulus of the volar half of the trapezium found in this study when looking at the advanced-stage OA group only. Inversely, a positive correlation was seen between VLC stiffness and permeability of the same area of the trapezium within the advanced-stage OA group. The attenuation of the VLC likely caused an increase in translation of the metacarpal on the trapezium [14] resulting in cartilage wear patterns seen in this study. SEM/EDS images showed a degenerating cartilage eventually resulting in eburnation of a majority of the volar half of the trapezium, similar to what previous studies have seen [17]. This is also further supported by the volume calculations from our MRI data. This eburnation results in an increase in equilibrium modulus as these areas transition from cartilage to eburnated subchondral bone. We surprisingly did not see a correlation between VLC stiffness and the biphasic properties of the volar portion of the metacarpal, despite the presence of metacarpal articular cartilage wear documented in this study and other previously published works [16, 17, 20, 21]. This could be explained by the trapezium surface being more sensitive to changes in articular cartilage than that of the metacarpal. Our SEM/EDS data showed that even in the earliest stages of OA, the volar central region of the trapezium was experiencing the beginnings of articular cartilage degeneration while the volar sections of cartilage in the metacarpal remained smooth and healthy. Additionally, we observed eburnation to be more severe in more regions within the trapezium than within the metacarpal. The trapezium had more specimens in more regions that were fully eburnated within the advanced-OA group. While the metacarpal only demonstrated full cartilage eburnation within the volar ulnar region in advanced-stage OA specimens, the trapezium demonstrated full eburnation in the volar radial, volar central, volar ulnar, dorsal central, and dorsal ulnar regions in certain advanced-stage OA specimens. It is important to note that the volar ulnar region of the metacarpal did have more partial eburnation (meaning spots of eburnated bone adjacent to areas of cartilage), but the eburnation within these regions was often concentrated directly adjacent to the intraarticular attachment of the beak ligament and did not spread into the full volar ulnar region. This type of pattern is supported in other studies that show cartilage wear and contact areas to be more concentrated within the volar ulnar region of the metacarpal while being more dispersed within the trapezium [12, 14, 21].

Since the VLC is a critical stabilizer of the TMC joint, with its deep layer functioning as a hinge point, its integrity plays a pivotal role in joint stability [12, 14, 20, 23, 31, 32]. The VLC attaches from the volar side of the trapezium to the base of the first metacarpal and is particularly important in resisting dorsoradial subluxation while maintaining joint congruency during thumb motion [9, 23, 32, 33]. Attrition of the VLC may compromise this stabilizing function, leading to joint instability characterized by abnormal palmar-dorsal and ulnar-radial translations [14, 34]. These altered kinematics could result in focal cartilage wear, specifically at the volar ulnar portion of the metacarpal and the volar central and ulnar regions of the trapezium. Further characterization of these kinematic changes is warranted to better understand the pathomechanics of TMC joint degeneration.

The areas with the most prominent changes in equilibrium modulus within the TMC joint included the volar ulnar region of the metacarpal and the volar central and ulnar regions of the trapezium. The increase in equilibrium modulus of these regions in osteoarthritic specimens can likely be explained by the increase in cartilage eburnation that was localized to these areas. Our SEM/EDS images showed severe cartilage eburnation, represented by large amounts of phosphorous and calcium coupled with little to no amounts of carbon, specific to these regions, further supporting the increase in equilibrium modulus. This most significant change occurred between the younger healthy/early-stage OA group and advanced-stage osteoarthritic group. We did see weaker, less significant, trends in the same regions when comparing the elder healthy/early-stage OA group to the advanced-stage OA group. The SEM/EDS images for the regions within the elder specimens reveals a rougher, more hole filled articular cartilage, as well as some spots of what appears to be the beginning process of eburnation. Since the tissue quality is already deteriorating in this group, there is not as much change between it and the advanced-stage OA group as what was seen between the, often smooth, regions in the younger group.

The absence of change in permeability values from group to group is likely due to a lack of sensitivity in the curve-fitting process specific to these samples. In the curve-fitting, equilibrium modulus was based on a single point where the sample reaches equilibrium while permeability was dependent on the entire dataset. For our data, we found that the predicted curve was well fit at the point of equilibrium in all samples and well fit for the entire dataset in a majority of our samples. However, in some of the regions that showed more severe cartilage degeneration or eburnation, the predicted curve did not fit the dataset very well in its entirety. This is most likely due to the contact radius being greater than 55% of cartilage thickness since the thickness of articular cartilage in osteoarthritic TMC joints does decrease, as supported by this study. This resulted in having to drop some permeability data points in the more affected regions, as the predicted deformation curve calculated was not close enough to the actual deformation (r^2^ < 0.70). We were able to keep every equilibrium modulus data point as the equilibrium portion of the curve was always accurately fit.

While this study was able to provide insight into the regional specific changes of TMC articular cartilage throughout OA progression and how ligament attenuation correlates to those regional changes, some limitations were still present. In many of the advanced-stage OA sample and some elder healthy/early-stage specimens, there was very little or no cartilage in certain regions, specifically the volar ulnar region of the metacarpal and the volar and dorsal central regions. This not only made it difficult to calculate permeability, as previously mentioned, but also could explain the high variability and lack of significance seen in the porosity results. Some samples’ wet weight was as small as 0.6 mg. When a sample of this size was weighed in PBS, the scale may not have been sensitive enough to accurately record this weight needed for porosity measurements. Additionally, due to the small size of the cartilage samples, GAG assays were not performed because the dry weight of many of the advanced-stage OA and elder healthy/early-stage OA specimens did not meet the minimum dry weight needed for the assay.

Additionally, we did not see any differences in biphasic properties between the younger and elder healthy/early-stage groups in any regions. This is likely due to these groups being based on both age and Eaton-Littler grade as opposed to being based exclusively on the Eaton-Littler grade. Our study shows that some of the elder healthy/early-stage OA samples already have eburnation present in some regions, which would raise the average equilibrium modulus values and lower permeability values, preventing us from seeing the decreased equilibrium modulus and increased permeability that is common in osteoarthritic articular cartilage [35–37]. Utilizing the Eaton-Littler classification system to separate specimens into disease groups could also contributed to the lack of change between the younger and elder healthy/early-stage OA groups.

The Eaton-Littler system relies on radiographs, meaning it does not take into consideration articular cartilage thickness or integrity. Increasing the sample size and adding presence of eburnation as a new parameter for sorting samples into disease groups could aid us in seeing a more significant difference between disease group. By adding this new parameter, it could also aid us in improving the correlation between VLC stiffness and the biphasic properties of articular cartilage in the volar ulnar region of the metacarpal. Increasing the sample size, specifically regarding mid to advanced-stage specimens that are only partially eburnated, may help us in further understanding this transition from healthy cartilage to attenuated cartilage to eburnated cartilage.

In conclusion, the volar ulnar region of the metacarpal and volar central and volar ulnar regions of the trapezium display(s) an increase in equilibrium modulus in advanced-stage OA TMC joints as the surface transitions from healthy cartilage to eburnated bone, which can be further supported by our SEM/EDS and MRI data. The changes seen within the articular cartilage of the trapezium, the more sensitive to wear of the two surfaces, can be correlated to the attenuation of the VLC within the same female TMC joints.

## Supplementary Information

Below is the link to the electronic supplementary material.

Supplementary file1 (PDF 2585 KB)

Supplementary file2 (PDF 964 KB)
