# Supplementary material for: A Regional Mapping of Articular Cartilage Integrity and Biphasic Properties in Healthy and Osteoarthritic Trapeziometacarpal Joints
Source: Ann Biomed Eng. 2025 Apr 2;53(6):1471–85. doi: 10.1007/s10439-025-03726-x (PMC12075277; doi:10.1007/s10439-025-03726-x)
Supplement: Supplementary file 1 — Supplementary file1 (PDF 2585 KB) [file 10439_2025_3726_MOESM1_ESM.pdf]

Online Resource 1

A Regional Mapping of Articular Cartilage Integrity and Biphasic Properties in Healthy and Osteoarthritic Trapeziometacarpal Joints

Lizzie Walker[1] (mewlkr@g.clemson.edu, ORCiD: 0009-0001-9980-6260), Hui Li[1] (hui3@g.clemson.edu), Nathan Buchweitz[1] (nbuchwe@g.clemson.edu), Daniel Gordon[1] (digordo@g.clemson.edu), Shangping Wang[1,2] (shagpw@clemson.edu ORCiD: 0000-0003-0049-2212), Dane Daley[2] (dalda@musc.edu), Hai Yao[1,2] (haiyao@clemson.edu), Yongren Wu[1,2] (yongren@clemson.edu ORCiD: 0000-0002-5411-8528)

Author Affiliations:

(1) Department of Bioengineering, Clemson University, Clemson, SC

(2) Department of Orthopaedics and Physical Medicine, Medical University of South Carolina, Charleston, SC

Corresponding Author: Corresponding Author: Yongren Wu, yongren@clemson.edu, 843-876-2305, 68 President Street, BEB 203, Charleston, SC, 29425

Outcome	Interaction Significance				
	OA Group	Region	Ligament	OA Group*Region	OA Group*Ligament
**Thickness**	<0.001	<0.001		0.003	
**Equilibrium Modulus**	0.023	0.173		0.508	
**Permeability**	0.297	0.017		0.755	
**Porsity**	0.292	0.782		0.051	
**Water Content**	0.252	0.869		0.543	
**Articular Cartilage Volume**	0.116	<0.001		0.210	
**Articular Cartilage Surface Area**	0.063	0.003		0.485	
**Ligament Volume**	0.218		0.014		0.257
**Ligament Surface Area**	0.235		0.008		0.338

Supplemental Table 1. Interaction significance of variables on each outcome.

		Thickness (mm)	Permeability ($x10^{-15}$ m^4/Ns)	Equilibrium Modulus (Mpa)	Porosity	Water Content (%)	Articular Cartilage Volume (MRI) (mm^3)	Articular Cartilage Surface Area (MRI) (mm^2)
Younger Healthy/Early-Stage OA	MC DR	0.67 ± 0.04	0.98 ± 0.23	0.49 ± 0.06	0.83 ± 0.03	68.81 ± 5.44	18.63 ± 1.44	66.50 ± 3.44
	MC DC	0.76 ± 0.02	0.90 ± 0.07	0.58 ± 0.05	0.84 ± 0.06	67.45 ± 4.38	20.87 ± 2.42	71.08 ± 6.96
	MC DU	0.83 ± 0.05	1.13 ± 0.16	0.41 ± 0.06	0.71 ± 0.04	60.71 ± 3.69	19.82 ± 3.94	74.58 ± 13.47
	MC VR	0.56 ± 0.05	0.87 ± 0.18	0.34 ± 0.04	0.74 ± 0.02	66.18 ± 1.86	18.52 ± 1.46	67.44 ± 7.55
	MC VC	0.62 ± 0.05	0.98 ± 0.25	0.33 ± 0.06	0.79 ± 0.01	70.51 ± 1.14	19.89 ± 1.82	67.67 ± 1.13
	MC VU	0.69 ± 0.07	1.14 ± 0.12	0.29 ± 0.03	0.78 ± 0.05	65.29 ± 4.66	18.11 ± 2.64	63.38 ± 4.70
	TR DR	0.72 ± 0.05	0.83 ± 0.10	0.46 ± 0.09	0.74 ± 0.06	62.91 ± 4.23	18.25 ± 2.04	64.51 ± 6.73
	TR DC	0.74 ± 0.07	1.10 ± 0.14	0.66 ± 0.09	0.75 ± 0.08	64.89 ± 6.07	18.80 ± 1.11	69.75 ± 3.96
	TR DU	0.66 ± 0.08	0.78 ± 0.08	0.42 ± 0.02	0.63 ± 0.07	56.22 ± 6.90	18.23 ± 2.80	68.18 ±9.27
	TR VR	0.72 ± 0.07	1.43 ± 0.27	0.39 ± 0.07	0.88 ± 0.05	72.38 ± 4.36	20.63 ± 1.97	71.73 ± 5.02
	TR VC	0.72 ± 0.02	1.02 ± 0.18	0.64 ± 0.09	0.75 ± 0.04	66.34 ± 3.21	18.97 ± 1.86	65.08 ± 3.92
	TR VU	0.83 ± 0.08	1.18 ± 0.23	0.42 ± 0.09	0.81 ± 0.03	71.05 ± 4.54	15.95 ± 2.26	65.87 ± 7.31
Elder Healthy/Early-Stage OA	MC DR	0.72 ± 0.08	0.92 ± 0.10	0.39 ± 0.08	0.71 ± 0.06	57.65 ± 5.45	27.04 ± 5.89	87.11 ± 17.83
	MC DC	0.76 ± 0.10	1.13 ± 0.22	0.52 ± 0.11	0.81 ± 0.04	69.46 ± 2.78	19.69 ± 4.25	79.25 ± 15.50
	MC DU	0.80 + 0.07	1.60 ± 0.20	0.40 ± 0.04	0.87 ± 0.03	73.00 ± 1.82	21.43 ± 6.47	81.96 ± 22.42
	MC VR	0.76 ± 0.08	1.48 ± 0.23	0.27 ± 0.04	0.79 ± 0.03	63.71 ± 4.20	17.63 ± 1.19	59.96 ± 4.34
	MC VC	0.63 ± 0.06	1.33 ± 0.22	0.41 ± 0.05	0.78 ± 0.02	67.18 ± 2.39	12.32 ± 2.58	54.55 ± 9.37
	MC VU	0.68 ± 0.06	1.47 ± 0.19	0.39 ± 0.04	0.80 ± 0.04	70.33 ± 3.32	10.99 ± 3.16	46.33 ± 10.60
	TR DR	0.81 ± 0.06	1.17 ± 0.20	0.36 ± 0.03	0.85 ± 0.04	71.10 ± 7.50	16.94 ± 0.83	47.79 ± 11.11
	TR DC	0.63 ± 0.06	1.35 ± 0.19	0.37 ± 0.06	0.84 ± 0.03	74.55 ± 2.53	17.18 ± 2.22	49.62 ± 9.53
	TR DU	0.66 ± 0.08	1.23 ± 0.23	0.37 ± 0.06	0.83 ± 0.06	66.32 ± 6.65	17.39 ± 5.86	47.57 ± 15.73
	TR VR	0.75 ± 0.08	1.48 ± 0.20	0.34 ± 0.05	0.71 ± 0.21	59.28 ± 7.89	15.81 ± 2.44	47.78 ± 13.10
	TR VC	0.71 ± 0.08	1.27 ± 0.28	0.44 ± 0.05	0.77 ± 0.04	66.48 ± 3.91	11.05 ± 1.61	40.80 ± 11.19
	TR VU	0.86 ± 0.07	0.88 ± 0.09	0.43 ± 0.08	0.76 ± 0.04	68.59 ± 4.88	11.25 ± 2.36	42.85 ± 14.16
Advanced-Stage OA	MC DR	0.65 ± 0.04	1.30 ± 0.31	0.40 ± 0.09	0.77 ± 0.07	70.75 ± 5.19	19.27 ± 1.17	68.64 ± 4.66
	MC DC	0.69 ± 0.08	1.28 ± 0.26	0.52 ± 0.11	0.83 ± 0.07	72.33 ± 4.98	17.18 ± 3.58	64.01 ± 11.67
	MC DU	0.54 ± 0.12	1.50 ± 0.25	0.43 ± 0.12	0.74 ± 0.04	63.95 ± 4.62	16.84 ± 2.81	60.32 ± 10.13
	MC VR	0.62 ± 0.12	1.38 ± 0.12	0.45 ± 0.17	0.82 ± 0.04	75.73 ± 4.00	15.05 ± 3.97	57.67 ± 9.45
	MC VC	0.48 ± 0.07	1.32 ± 0.16	0.48 ± 0.16	0.80 ± 0.02	70.77 ± 0.96	10.58 ± 2.62	49.43 ± 7.72
	MC VU	0.25 ± 0.05	1.08 ± 0.26	0.83 ± 0.29	0.80 ± 0.03	67.44 ± 2.25	7.69 ± 1.17	39.25 ± 5.54
	TR DR	0.46 ± 0.12	0.83 ± 0.05	0.49 ± 0.06	0.86 ± 0.06	70.05 ± 6.69	12.38 ± 3.31	57.98 ± 2.58
	TR DC	0.17 ± 0.03	1.10 ± 0.01	0.83 ± 0.26	0.88 ± 0.04	82.44 ± 7.81	16.05 ± 2.50	59.95 ± 6.82
	TR DU	0.41 ± 0.12	0.98 ± 0.17	0.48 ± 0.32	0.81 ± 0.03	71.29 ± 6.32	20.50 ± 3.35	75. 55 ± 6.91
	TR VR	0.47 ± 0.05	1.18 ± 0.16	0.86 ± 0.33	0.67 ± 0.08	60.49 ± 7.33	7.29 ± 1.71	37.90 ± 6.64
	TR VC	0.24 ± 0.07	0.87 ± 0.12	1.13 ± 0.29	0.84 ± 0.01	75.50 ± 0.50	5.99 ± 3.28	28.55 ± 13.75
	TR VU	0.44 ± 0.13	0.88 ± 0.11	0.99 ± 0.51	0.81 ± 0.02	71.38 ± 0.82	10.10 ± 4.60	41.29 ± 14.95

		Ligament Volume (MRI) (mm^3)	Ligament Surface Area (MRI) (mm^2)
Younger Healthy/Early-Stage OA	VLC	56.77 ± 18.54	185.64 ± 49.48
	DRL	61.35 ± 7.75	214.14 ± 23.94
Elder Healthy/Early-Stage OA	VLC	64.25 ± 15.21	210.34 ± 37.77
	DRL	105.26 ± 11.34	312.93 ± 25.30
Advanced-Stage OA	VLC	53.19 ± 9.66	180.11 ± 18.25
	DRL	107.82 ± 22.67	318.28 ± 52.19

Supplemental Table 2. Mean and standard error for each outcome. N = 6 for thickness, permeability, equilibrium modulus, porosity, and water content. N = 4 for MRI measurements.

Thickness										
YH vs EH Comparisons				YH vs OA Comparisons				EH vs OA Comparisons		
**Region**	**p. adj.**	**Mean diff (95% CI)**		**Region**	**p. adj.**	**Mean diff (95% CI)**		**Region**	**p. adj.**	**Mean diff (95% CI)**
MC DR	1	-0.061 (-0.307, 0.186)		MC DR	1	0.054 (-0.195, 0.302)		MC DR	0.801	0.114 (-0.134, 0.363)
MC DC	1	-0.012 (-0.259, 0.234)		MC DC	1	0.069 (-0.179, 0.317)		MC DC	1	0.081 (-0.167, 0.330)
MC DU	1	0.025 (-0.222, 0.272)		MC DU	**0.009***	0.311 (0.062, 0.559)		MC DU	**0.018***	0.286 (0.038, 0.534)
MC VR	0.35	-0.161 (-0.408, 0.086)		MC VR	1	-0.068 (-0.317, 0.181)		MC VR	1	0.093 (-0.156, 0.343)
MC VC	1	0.027 (-0.220, 0.274)		MC VC	0.59	0.134 (-0.116, 0.383)		MC VC	0.913	0.106 (-0.143, 0.355)
MC VU	1	0.046 (-0.201, 0.293)		MC VU	**<0.001***	0.433 (0.184, 0.682)		MC VU	**<0.001***	0.387 (0.138, 0.636)
TR DR	1	-0.060 (-0.307, 0.186)		TR DR	**0.046***	0.252 (0.004, 0.500)		TR DR	**0.008***	0.312 (0.064, 0.560)
TR DC	1	0.063 (-0.183, 0.310)		TR DC	**<0.001***	0.513 (0.265, 0.762)		TR DC	**<0.001***	0.450 (0.202. 0.699)
TR DU	1	-0.001 (-0.247, 0.245)		TR DU	**0.088**	0.226 (-0.023, 0.474)		TR DU	**0.086**	0.227 (-0.022, 0.475)
TR VR	1	-0.028 (-0.275, 0.218)		TR VR	**0.083**	0.228 (-0.021, 0.478)		TR VR	**0.041***	0.257 (0.008, 0.506)
TR VC	1	0.013 (-0.233, 0.260)		TR VC	**<0.001***	0.468 (0.219, 0.717)		TR VC	**<0.001***	0.455 (0.205, 0.704)
TR VU	1	-0.051 (-0.297, 0.196)		TR VU	**0.001***	0.373 (0.124, 0.622)		TR VU	**<0.001***	0.423 (0.174, 0.672)

Supplemental Table 3. Comparison of articular cartilage thickness for each region between disease group derived from linear mixed effects model.

Region Comparisons in OA Group for Thickness											
	**MC DC**	**MC DU**	**MC VR**	**MC VC**	**MC VU**	**TR DR**	**TR DC**	**TR DU**	**TR VR**	**TR VC**	**TR VU**
**MC DR**	1<br>-0.067 (-0.381, 0.246)	1 0.066<br>(-0.251, 0.384)	1 -<br>0.020 (-0.339, 0.299)	1 0.117 (-<br>0.201, 0.436)	**0.017***<br>0.347 (0.028, 0.666)	1<br>0.144 (-0.173, 0.461)	**0.002***<br>0.405 (0.084, 0.726)	1<br>0.166 (-0.155, 0.488)	1 0.125<br>(-0.194, 0.443)	**0.012***<br>0.356 (0.038, 0.675)	1<br>0.160 (-0.158, 0.479)
**MC DC**		1<br>0.134 (-0.181, 0.448)	1<br>0.047 (-0.275, 0.370)	1<br>0.185 (-0.138, 0.507)	**0.001***<br>0.414 (0.092, 0.737)	1<br>0.212 (-0.105, 0.529)	**<0.001***<br>0.473 (0.154, 0.791)	0.843<br>0.234 (-0.085, 0.552)	1<br>0.192 (-0.130, 0.514)	**<0.001***<br>0.424 (0.102, 0.746)	1<br>0.228 (-0.094, 0.550)
**MC DU**			1<br>-0.086 (-0.404, 0.232)	1<br>0.051 (-0.267, 0.369)	0.187<br>0.281 (-0.037, 0.599)	1<br>0.078 (-0.242, 0.398)	**0.02***<br>0.339 (0.024, 0.653)	1<br>0.100 (-0.214, 0.415)	1<br>0.058 (-0.259, 0.376)	0.135<br>0.290 (-0.028, 0.608)	1<br>0.094 (-0.224, 0.412)
**MC VR**				1<br>0.137 (-0.173, 0.448)	**0.005***<br>0.367 (0.056, 0.678)	1<br>0.165 (-0.153, 0.482)	**<0.001*** 0.425<br>(0.111, 0.739)	1<br>0.186 (-0.127, 0.500)	1<br>0.145 (-0.166, 0.456)	**0.003***<br>0.377 (0.066, 0.687)	1<br>0.180 (-0.130, 0.491)
**MC VC**					0.796<br>0.230 (-0.081, 0.540)	1<br>0.027 (-0.290, 0.345)	0.128<br>0.288 (-0.026, 0.602)	1<br>0.049 (-0.265, 0.363)	1<br>0.007 (-0.303, 0.318)	0.594<br>0.239 (-0.072, 0.550)	1<br>0.043 (-0.268, 0.354)
**MC VU**						1<br>-0.202 (-0.520, 0.115)	1<br>0.058 (-0.256, 0.372)	1<br>-0.181 (-0.494, 0.133)	0.996<br>-0.222 (-0.533, 0.089)	1<br>0.009 (-0.301, 0.320)	1<br>-0.187 (-0.497, 0.124)
**TR DR**							0.349<br>0.261 (-0.056, 0.577)	1<br>0.022 (-0.295, 0.339)	1<br>-0.020 (-0.337, 0.298)	1<br>0.212 (-0.105, 0.529)	1<br>0.016 (-0.301, 0.333)
**TR DC**								0.601<br>-0.239 (-0.550, 0.072)	0.167<br>-0.280 (-0.594, 0.033)	1<br>-0.049 (-0.363, 0.265)	0.539<br>-0.245 (-0.559, 0.069)
**TR DU**									1<br>-0.042 (-0.355, 0.272)	1<br>0.190 (-0.124, 0.504)	1<br>-0.006 (-0.320, 0.308)
**TR VR**										0.749<br>0.232 (-0.079, 0.542)	1<br>0.036 (-0.275, 0.346)
**TR VC**											1<br>-0.196 (-0.507, 0.115)
**TR VU**											

Supplemental Table 4. Comparison of articular cartilage thickness between regions in the advanced-stage OA group derived from linear mixed effects model.

Equilibrium Modulus										
YH vs EH Comparisons				YH vs OA Comparisons				EH vs OA Comparisons		
**Region**	**p. adj.**	**Mean diff (95% CI)**		**Region**	**p. adj.**	**Mean diff (95% CI)**		**Region**	**p. adj.**	**Mean diff (95% CI)**
MC DR	1	0.064 (-0.452, 0.580)		MC DR	1	0.086 (-.0432, 0.603)		MC DR	1	0.022 (-0.496, 0.539)
MC DC	1	0.044 (-0.471, 0.560)		MC DC	1	0.058 (-0.459, 0.575)		MC DC	1	0.014 (-0.504, 0.531)
MC DU	1	0.017 (-0.499, 0.533)		MC DU	1	-0.052 (-0.570, 0.465)		MC DU	1	-0.069 (-0.587, 0.448)
MC VR	1	0.067 (-0.450, 0.583)		MC VR	1	-0.110 (-0.629, 0.408)		MC VR	1	-0.177 (-0.696, 0.342)
MC VC	1	-0.091 (-0.607, 0.426)		MC VC	1	-0.192 (-0.710, 0.327)		MC VC	1	-0.101 (-0.620, 0.418)
MC VU	1	-0.116 (-0.633, 0.401)		MC VU	**0.024***	-0.574 (-1.093, -0.056)		MC VU	**0.102**	-0.458 (-0.977, 0.060)
TR DR	1	0.099 (-0.416, 0.614)		TR DR	1	-0.022 (-0.520, 0.515)		TR DR	1	-0.101 (-0.619, 0.416)
TR DC	0.625	0.269 (-0.246, 0.785)		TR DC	1	-0.134 (-0.652, 0.384)		TR DC	0.184	-0.404 (-0.921, 0.114)
TR DU	1	0.047 (-0.468, 0.563)		TR DU	0.288	-0.359 (-0.876, 0.159)		TR DU	0.179	-0.406 (-0.924, 0.112)
TR VR	1	0.037 (-0.479, 0.553)		TR VR	0.141	-0.429 (-0.947, 0.089)		TR VR	**0.093**	-0.466 (-0.984, 0.052)
TR VC	1	0.183 (-0.333, 0.699)		TR VC	**0.092**	-0.467 (-0.985, 0.051)		TR VC	**0.008***	-0.650 (-1.169, -0.132)
TR VU	1	-0.035 (-0.551, 0.480)		TR VU	**0.037***	-0.543 (-1.062, -0.025)		TR VU	**0.05***	-0.508 (-1.026, 0.010)

Supplemental Table 5. Comparison of equilibrium modulus for each region between disease group derived from linear mixed effects model.

Permeability										
YH vs EH Comparisons				YH vs OA Comparisons				EH vs OA Comparisons		
Region	p. adj.	Mean diff (95% CI)		Region	p. adj.	Mean diff (95% CI)		Region	p. adj.	Mean diff (95% CI)
MC DR	1	0.111 (-1.079, 1.301)		MC DR	1	-0.262 (-1.454, 0.931)		MC DR	1	-0.373 (-1.566, 0.820)
MC DC	1	-0.077 (-1.266, 1.113)		MC DC	1	-0.258 (-1.450, 0.934)		MC DC	1	-0.181 (-1.373, 1.011)
MC DU	1	-0.477 (-1.667, 0.713)		MC DU	1	-0.402 (-1.594, 0.791)		MC DU	1	0.075 (-1.117, 1.267)
MC VR	1	-0.448 (-1.638, 0.743)		MC VR	1	-0.366 (-1.615,0.883)		MC VR	1	0.082 (-1.168, 1.331)
MC VC	1	-0.315 (-1.506, 0.876)		MC VC	1	-0.287 (-1.537, 0.963)		MC VC	1	0.028 (-1.221, 1.278)
MC VU	1	2.538 (1.347, 3.729)		MC VU	1	2.816 (1.487, 4.144)		MC VU	1	0.278 (-1.050, 1.606)
TR DR	1	-0.304 (-1.493, 0.885)		TR DR	1	-0.149 (-1.341, 1.043)		TR DR	1	0.155 (-1.037, 1.348)
TR DC	1	-0.126 (-1.371, 1.120)		TR DC	1	-0.001 (-1.379, 1.376)		TR DC	1	0.124 (-1.203, 1.452)
TR DU	1	-0.440 (-1.630, 0.749)		TR DU	1	-0.141 (-1.390, 1.107)		TR DU	1	0.299 (-0.949, 1.547)
TR VR	1	0.144 (-1.045, 1.334)		TR VR	1	0.342 (-0.985, 1.670)		TR VR	1	0.198 (-1.130, 1.526)
TR VC	1	-0.290 (-1.480, 0.901)		TR VC	1	0.004 (-1.444, 1.452)		TR VC	1	0.294 (-1.155, 1.742)
TR VU	1	0.388 (-0.802, 1.577)		TR VU	1	0.278 (-0.971, 1.527)		TR VU	1	-0.109 (-1.358, 1.139)

Supplemental Table 6. Comparison of permeability for each region between disease group derived from linear mixed effects model.

Correlations							
Comparisons			r	r^2	p value	Surface	Disease Group
VLC Stiffness	vs	Permeability	0.0308	0.001	0.9283	MC	YH
VLC Stiffness	vs	Equilibrium Modulus	0.2462	0.0606	0.4656	MC	YH
VLC Stiffness	vs	Permeability	0.1272	0.0162	0.6789	MC	EH
VLC Stiffness	vs	Equilibrium Modulus	-0.0668	0.0045	0.8283	MC	EH
VLC Stiffness	vs	Permeability	-0.3705	0.1373	0.292	MC	OA
VLC Stiffness	vs	Equilibrium Modulus	0.1201	0.0144	0.7101	MC	OA
VLC Stiffness	vs	Permeability	0.4381	0.1919	0.1543	TR	YH
VLC Stiffness	vs	Equilibrium Modulus	0.1791	0.0321	0.5776	TR	YH
VLC Stiffness	vs	Permeability	-0.1126	0.0127	0.7015	TR	EH
VLC Stiffness	vs	Equilibrium Modulus	-0.2253	0.0507	0.4387	TR	EH
VLC Stiffness	vs	Permeability	0.7795	0.6076	**0.0226***	TR	OA
VLC Stiffness	vs	Equilibrium Modulus	-0.7051	0.4971	**0.0049***	TR	OA

Supplemental Table 7. Pearson correlations between VLC stiffness obtained through load-to-failure testing [15] and biphasic properties of the palmar half of the metacarpal and trapezium surfaces from the same cohort of female samples.

Correlations							
Comparisons			r	r^2	p value	Surface	Disease Group
DRL Stiffness	vs	Permeability	-0.2011	0.0405	0.5531	MC	YH
DRL Stiffness	vs	Equilibrium Modulus	-0.6534	0.4269	0.0293	MC	YH
DRL Stiffness	vs	Permeability	-0.1112	0.0124	0.7308	MC	EH
DRL Stiffness	vs	Equilibrium Modulus	-0.3751	0.1407	0.2295	MC	EH
DRL Stiffness	vs	Permeability	-0.7699	0.5928	**0.0056**	MC	OA
DRL Stiffness	vs	Equilibrium Modulus	0.9323	0.8692	**0***	MC	OA
DRL Stiffness	vs	Permeability	0.2831	0.0801	0.399	TR	YH
DRL Stiffness	vs	Equilibrium Modulus	0.0648	0.0042	0.8499	TR	YH
DRL Stiffness	vs	Permeability	-0.1151	0.0132	0.6713	TR	EH
DRL Stiffness	vs	Equilibrium Modulus	0.043	0.0019	0.8742	TR	EH
DRL Stiffness	vs	Permeability	-0.4029	0.1623	0.4284	TR	OA
DRL Stiffness	vs	Equilibrium Modulus	0.1885	0.0355	0.6272	TR	OA

Supplemental Table 8. Pearson correlations between DRL stiffness obtained through load-to-failure testing [15] and biphasic properties of the dorsal half of the metacarpal and trapezium surfaces from the same cohort of female samples. Significance likely seen on the metacarpal surface in the OA group due to slight changes in DRL's mechanical properties (not significant) and slight changes in dorsal articular cartilage (not significant).

Porosity										
YH vs EH Comparisons				YH vs OA Comparisons				EH vs OA Comparisons		
Region	p. adj.	Mean diff (95% CI)		Region	p. adj.	Mean diff (95% CI)		Region	p. adj.	Mean diff (95% CI)
MC DR	0.389	0.119 (-0.070, 0.308)		MC DR	1	0.064 (-0.109, 0.237)		MC DR	1	-0.055 (-0.228, 0.118)
MC DC	1	0.026 (-0.143, 0.195)		MC DC	1	0.004 (-0.176, 0.183)		MC DC	1	-0.022 (-0.202, 0.157)
MC DU	**0.067**	-0.155 (-0.317, 0.007)		MC DU	1	-0.030 (-0.192, 0.132)		MC DU	0.288	0.125 (-0.044, 0.294)
MC VR	1	-0.050 (-0.223, 0.122)		MC VR	0.931	-0.079 (-0.269, 0.110)		MC VR	1	-0.029 (-0.202, 0.144)
MC VC	1	0.008 (-0.165, 0.181)		MC VC	1	-0.011 (-0.242, 0.221)		MC VC	1	-0.019 (-0.237, 0.200)
MC VU	1	-0.028 (-0.190, 0.134)		MC VU	1	-0.021 (-0.200, 0.159)		MC VU	1	0.007 (-0.166, 0.180)
TR DR	0.396	-0.112 (-0.292, 0.067)		TR DR	0.417	-0.116 (-0.305, 0.073)		TR DR	1	-0.004 (-0.183, 0.176)
TR DC	0.53	-0.097 (-0.269, 0.076)		TR DC	0.495	-0.133 (-0.365, 0.098)		TR DC	1	-0.037 (-0.255, 0.182)
TR DU	**0.032***	-0.192 (-0.372, -0.013)		TR DU	**0.048***	-0.180 (-0.360, -0.001)		TR DU	1	0.012 (-0.158, 0.181)
TR VR	0.969	0.066 (-0.096, 0.228)		TR VR	0.236	-0.019 (-0.199, 0.160)		TR VR	0.693	-0.086 (-0.258, 0.087)
TR VC	1	-0.017 (-0.197, 0.162)		TR VC	0.943	-0.093 (-0.317, 0.131)		TR VC	1	-0.076 (-0.308, 0.156)
TR VU	1	0.044 (-0.128, 0.217)		TR VU	1	-0.014 (-0.203, 0.175)		TR VU	1	-0.058 (-0.231, 0.114)

Supplemental Table 9. Comparison of porosity for each region between disease group derived from linear mixed effects model.

Water Content								
YH vs EH Comparisons			YH vs OA Comparisons			EH vs OA Comparisons		
Region	p. adj.	Mean diff (95% CI)	Region	p. adj.	Mean diff (95% CI)	Region	p. adj.	Mean diff (95% CI)
MC DR	0.604	10.979 (-9.754, 31.712)	MC DR	1	-1.921 (-20.850, 17.007)	MC DR	0.302	12.900 (-31.827, 6.027)
MC DC	1	0.067 (-18.478, 18.611)	MC DC	1	-3.100 (-22.769, 16.570)	MC DC	1	-3.166 (-22.835, 16.503)
MC DU	0.308	-12.030 (-29.785, 5.725)	MC DU	1	-3.168 (-20.925, 14.588)	MC DU	0.746	8.861 (-9.683, 27.406)
MC VR	1	2.384 (-16.542, 21.310)	MC VR	0.765	-9.776 (-30.509, 10.957)	MC VR	0.365	-12.160 (-31.087, 6.768)
MC VC	1	3.233 (-15.694, 22.159)	MC VC	1	-0.348 (-25.739, 25.043)	MC VC	1	-3.581 (-27.520, 20.358)
MC VU	1	-0.778 (-17.07, 16.152)	MC VU	1	1.897 (-17.031, 20.825)	MC VU	1	2.675 (-16.252, 21.602)
TR DR	0.896	-8.463 (-28.132, 11.206)	TR DR	1	-7.342 (-28.076, 13.391)	TR DR	1	1.120 (-18.549, 20.790)
TR DC	1	-0.462 (-18.840, 17.916)	TR DC	0.28	-17.690 (-43.078, -7.698)	TR DC	0.233	-17.228 (-40.733, 6.278)
TR DU	0.247	-13.659 (-32.585, 5.268)	TR DU	0.194	-15.105 (-34.774, 4.565)	TR DU	1	-1.446 (-19.202, 16.310)
TR VR	0.227	13.116 (-4.639, 30.871)	TR VR	0.442	11.826 (-7.844, 31.495)	TR VR	1	-1.290 (-20.217, 17.637)
TR VC	1	-1.717 (-21.386, 17.952)	TR VC	1	-9.245 (-33.773, 15.283)	TR VC	1	-7.528 (-32.916, 17.860)
TR VU	0.94	7.897 (-11.029, 26.824)	TR VU	1	5.844 (-16.549, 28.237)	TR VU	1	-2.053 (-22.786, 18.679)

Supplemental Table 10. Comparison of water content for each region between disease group derived from linear mixed effects model.

Articular Cartilage Volume (MRI)								
YH vs EH Comparisons			YH vs OA Comparisons			EH vs OA Comparisons		
Region	p. adj.	Mean diff (95% CI)	Region	p. adj.	Mean diff (95% CI)	Region	p. adj.	Mean diff (95% CI)
MC DR	0.188	-8.414 (-19.324, 2.496)	MC DR	1	-0.642 (-11.552, 10.268)	MC DR	0.255	7.771 (-3.138, 18.681)
MC DC	1	1.179 (-9.731, 12.089)	MC DC	1	3.691 (-7.219, 14.601)	MC DC	1	2.511 (-8.398, 13.421)
MC DU	1	-1.602 (-12.512, 9.308)	MC DU	1	2.985 (-7.925, 13.895)	MC DU	0.917	4.587 (-6.323, 15.497)
MC VR	1	0.888 (-10.022, 11.798)	MC VR	0.931	3.468 (-7.442, 14.378)	MC VR	1	2.580 (-8.330, 13.490)
MC VC	0.279	7.572 (-3.338, 18.482)	MC VC	0.12	9.306 (-1.604, 20.216)	MC VC	1	1.734 (-9.176, 12.644)
MC VU	0.32	7.269 (-3.641, 18.179)	MC VU	**0.066**	10.421 (-0.489, 21.331)	MC VU	1	3.152 (-7.758, 14.062)
TR DR	1	-0.729 (-11.639, 10.181)	TR DR	0.573	5.870 (-5.040, 16.780)	TR DR	0.427	6.598 (-4.311, 17.508)
TR DC	1	-0.114 (-11.024, 10.796)	TR DC	1	2.742 (-8.168, 13.652)	TR DC	1	2.855 (-8.054, 13.765)
TR DU	1	0.843 (-10.067, -11.753)	TR DU	1	-2.269 (-13.179, 8.641)	TR DU	1	-3.112 (-14.022, 7.798)
TR VR	0.844	4.827 (-6.063, 15.737)	TR VR	**0.011***	13.348 (2.438, 24.558)	TR VR	0.178	8.521 (-2.389, 19.431)
TR VC	0.238	7.971 (-2.993, 18.827)	TR VC	**0.014***	12.979 (2.069, 23.889)	TR VC	0.776	5.061 (-5.848, 15.971)
TR VU	0.88	4.707 (-6.203, 15.617)	TR VU	0.575	5.858 (-6.052, 16.768)	TR VU	1	1.151 (-9.759, 12.061)

Supplemental Table 11. Comparison of MRI derived articular cartilage volume for each region between disease group derived from linear mixed effects model.

Articular Cartilage Surface Area (MRI)								
YH vs EH Comparisons			YH vs OA Comparisons			EH vs OA Comparisons		
Region	p. adj.	Mean diff (95% CI)	Region	p. adj.	Mean diff (95% CI)	Region	p. adj.	Mean diff (95% CI)
MC DR	0.481	-20.616 (-56.098, 14.866)	MC DR	1	-2.134 (-37.616, 33.349)	MC DR	0.623	18.483 (-17.000, 53.965)
MC DC	1	-8,672 (-44.154, 26.811)	MC DC	1	7.075 (-28.407, 42.557)	MC DC	0.848	15.747 (-19.736, 51.229)
MC DU	1	-7.377 (-42.859, 28.106)	MC DU	0.99	14.267 (-21.215, 49.749)	MC DU	0.422	21.643 (-13.839, 57.125)
MC VR	1	7.479 (-28.003, 42.961)	MC VR	1	9,769 (-25.713, 45.252)	MC VR	1	2.291 (-33.192, 37.773)
MC VC	1	13.124 (-22.358, 48.606)	MC VC	0.641	18.241 (-17.241, 53.723)	MC VC	1	5.117 (-30.365, 40.599)
MC VU	0.735	17.045 (-18.437, 52.527)	MC VU	0.303	24.126 (-11.356, 59.608)	MC VU	1	7.081 (-28.401, 42.563)
TR DR	0.735	17.041 (-18.441, 52.523)	TR DR	0.829	15.951 (-19.531, 51.433)	TR DR	1	-1.090 (-36.572, 34.393)
TR DC	0.528	19,869 (-15.613, 55.351)	TR DC	1	10.311 (-25.171, 45.793)	TR DC	1	-9.558 (-45.040, 25.924)
TR DU	0.481	20.610 (-14.872, 56.092)	TR DU	1	-7.368 (-42.851, 28.114)	TR DU	0.173	-27.978 (-63.460, 7.504)
TR VR	0.311	23.946 (-11.536, 59.428)	TR VR	**0.067**	33.828 (-1.684, 69.310)	TR VR	1	9.882 (-25.600, 45.364)
TR VC	0.297	24.277 (-11.205, 59.759)	TR VC	**0.041***	36.529 (1.046, 72.011)	TR VC	1	12.252 (-23.231, 47.734)
TR VU	0.352	23.018 (-12.464, 58.500)	TR VU	0.284	24.583 (-10.900, 60.065)	TR VU	1	1.564 (-33.918, 37.047)

Supplemental Table 12. Comparison of MRI derived articular cartilage surface area for each region between disease group derived from linear mixed effects model.

Ligament Volume (MRI)										
YH vs EH Comparisons				YH vs OA Comparisons				EH vs OA Comparisons		
Ligament	p. adj.	Mean diff (95% CI)		Region	p. adj.	Mean diff (95% CI)		Region	p. adj.	Mean diff (95% CI)
VLC	1	-7.487 (-63.903, 48.930)		VLC	1	3.576 (-52.841, 59.993)		VLC	1	11.063 (-45.354, 67.480)
DRL	1	-43.908 (-100.325, 12.509)		DRL	0.13	-46.466 (-102.883, 9.951)		DRL	0.164	-2.558 (-58.975, 53.859)

Supplemental Table 13. Comparison of MRI derived ligament volume for each ligament between disease group derived from linear mixed effects model.

Ligament Surface Area (MRI)										
YH vs EH Comparisons				YH vs OA Comparisons				EH vs OA Comparisons		
Ligament	p. adj.	Mean diff (95% CI)		Region	p. adj.	Mean diff (95% CI)		Region	p. adj.	Mean diff (95% CI)
VLC	1	-24.699 (-162.216, 112.819)		VLC	1	5.539 (-131.978, 143.057)		VLC	1	30.228 (-107.280, 167.756)
DRL	0.222	-98.786 (-236.304, 38.732)		DRL	0.183	-104.135 (-241.652, 33.383)		DRL	1	-5.348 (-142.866, 132.170)

Supplemental Table 14. Comparison of MRI derived ligament surface area for each ligament between disease group derived from linear mixed effects model.

Ligament Volume (MRI)		
VLC vs DRL Comparisons		
Disease Group	p. adj.	Mean diff (95% CI)
YH	0.833	-4.586 (-49.498, 40.325)
EH	**0.071**	-41.008 (-85.919, 3.903)
OA	**0.020***	-54.628 (-99.540, -9.717)

Supplemental Table 15. Comparison of MRI derived ligament volume between ligament type for each disease group derived from linear mixed effects model.

Ligament Surface Area (MRI)		
VLC vs DRL Comparisons		
Disease Group	p. adj.	Mean diff (95% CI)
YH	0.591	-28.496 (-137.968, 80.977)
EH	**0.065**	-102.584 (-212.056, 6.880)
OA	**0.016***	-138.170 (-247.642, -28.697)

Supplemental Table 16. Comparison of MRI derived ligament surface area between ligament type for each disease group derived from linear mixed effects model.
