# Supplementary material for: A Regional Mapping of Articular Cartilage Integrity and Biphasic Properties in Healthy and Osteoarthritic Trapeziometacarpal Joints
Source: Ann Biomed Eng. 2025 Apr 2;53(6):1471–85. doi: 10.1007/s10439-025-03726-x (PMC12075277; doi:10.1007/s10439-025-03726-x)
Supplement: Supplementary file 2 — Supplementary file2 (PDF 964 KB) [file 10439_2025_3726_MOESM2_ESM.pdf]

Online Resource 2

A Regional Mapping of Articular Cartilage Integrity and Biphasic Properties in Healthy and Osteoarthritic Trapeziometacarpal Joints

Lizzie Walker[1] (mewlkr@g.clemson.edu, ORCiD: 0009-0001-9980-6260), Hui Li[1] (hui3@g.clemson.edu), Nathan Buchweitz[1] (nbuchwe@g.clemson.edu), Daniel Gordon[1] (digordo@g.clemson.edu), Shangping Wang[1,2] (shagpw@clemson.edu ORCiD: 0000-0003-0049-2212), Dane Daley[2] (dalda@musc.edu), Hai Yao[1,2] (haiyao@clemson.edu), Yongren Wu[1,2] (yongren@clemson.edu ORCiD: 0000-0002-5411-8528)

Author Affiliations:

(1) Department of Bioengineering, Clemson University, Clemson, SC

(2) Department of Orthopaedics and Physical Medicine, Medical University of South Carolina, Charleston, SC

Corresponding Author: Corresponding Author: Yongren Wu, yongren@clemson.edu, 843-876-2305, 68 President Street, BEB 203, Charleston, SC, 29425

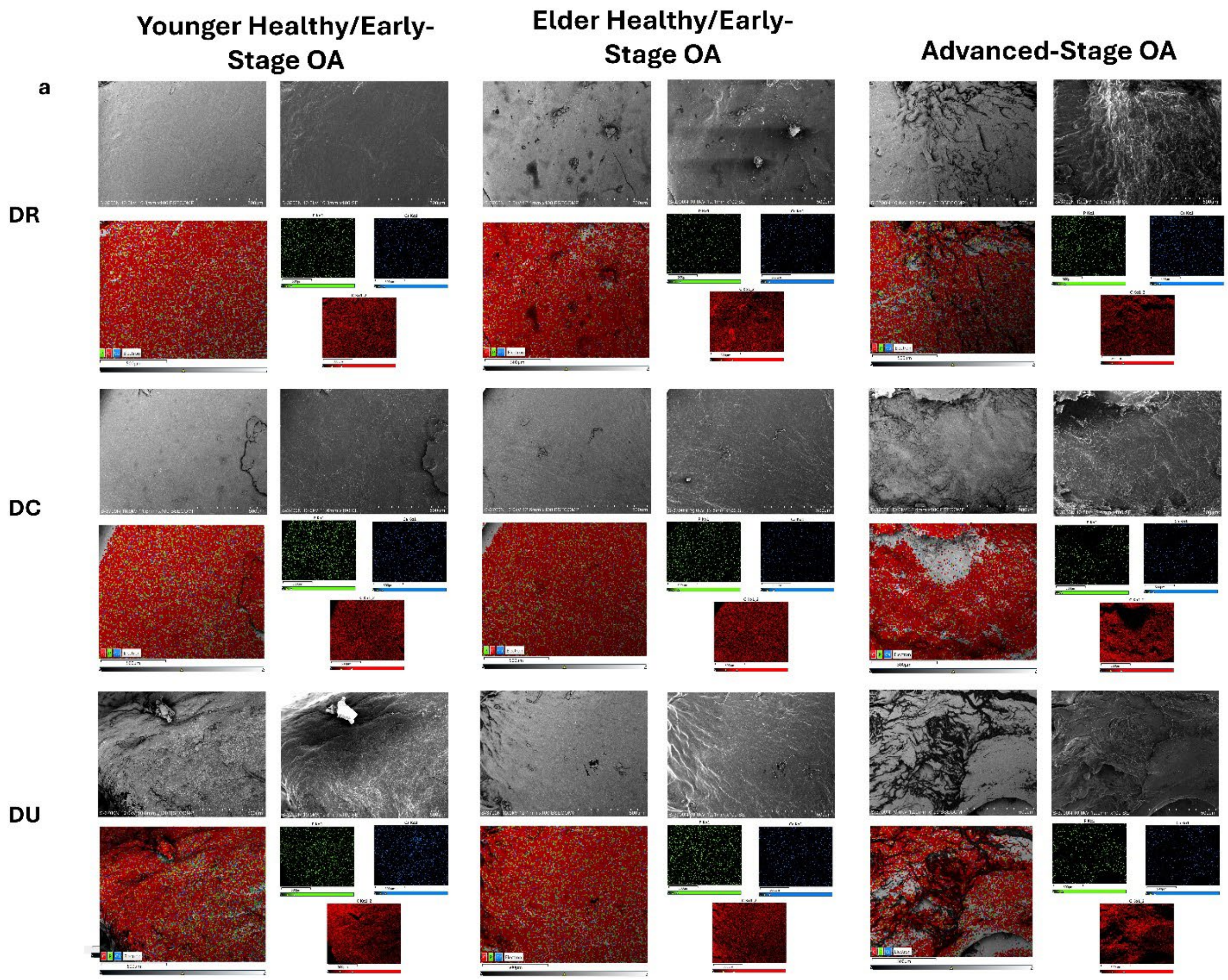
Younger Healthy/Early-Stage OA
Elder Healthy/Early-Stage OA
Advanced-Stage OA
a
DR
DC
DU

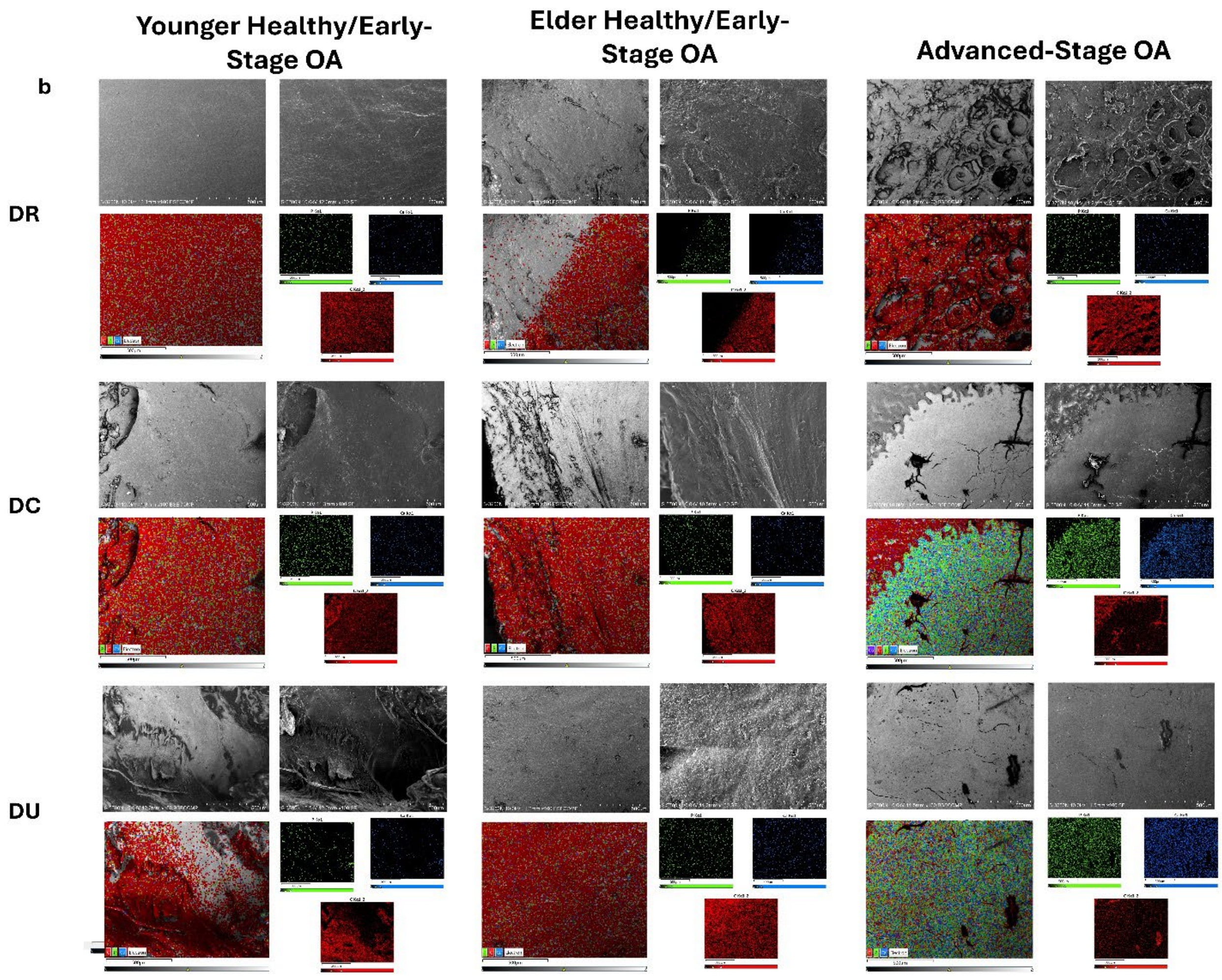

Supplemental Fig. 1: SEM/EDS images of representative samples from each disease group: **(a)** dorsal radial, dorsal central, and dorsal ulnar regions of the metacarpal **(b)** dorsal radial, dorsal central, and dorsal ulnar regions of the trapezium. Each region contains BSE image (top left), SE image (top right), EDS layered image (bottom left), and individual EDS layers (bottom right). Red represents carbon, green represents phosphorous, and blue represents calcium. Any gray areas on the EDS layered image are due to unevenness of the sample or shadows
